# Preparation and Characterization of Propolis–Biopolymer Coatings on Textiles for Topical Applications: Chemical Composition, Bioactivity, and Cytotoxicity Assessment

**DOI:** 10.3390/molecules31162746

**Published:** 2026-08-07

**Authors:** Iva Rezić Meštrović, Darinka Cvetković, Antonio Zandona, Maja Katalinić, Ernest Meštrović, Maja Somogyi Škoc

**Affiliations:** 1 Department of Applied Chemistry, Faculty of Textile Technology, University of Zagreb, Prilaz baruna Filipovića 28a, 10000 Zagreb, Croatia; 2 Department of Materials Testing, Faculty of Textile Technology, University of Zagreb, Prilaz baruna Filipovića 28a, 10000 Zagreb, Croatiamaja.somogyi@tt.unizg.hr (M.S.Š.); 3 Division of Toxicology, Institute for Medical Research and Occupational Health, Ksaverska cesta 2, 10001 Zagreb, Croatia; azandona@imi.hr (A.Z.); mkatalinic@imi.hr (M.K.); 4 Department of General and Inorganic Chemistry, Faculty of Chemical Engineering, University of Zagreb, Marulićev trg 19, 10000 Zagreb, Croatia; emestrov@fkit.unizg.hr

**Keywords:** propolis, coatings, honey-bee products, bioactivity, cytotoxicity, instrumental characterization, SEM, FTIR, UV-VIS, coffee-ring

## Abstract

Functional coatings based on natural bioactive compounds have attracted increasing interest in topical and biomedical applications. In this study, textile substrates were coated with formulations containing propolis and selected biopolymers to create biocompatible surfaces with enhanced physicochemical and biological properties. The coating preparation process was optimized to achieve uniform deposition and stable incorporation of bioactive constituents. The chemical composition and interactions between propolis, biopolymers, and textile fibers were investigated using Fourier-transform infrared spectroscopy (FTIR), while surface morphology and coating distribution were characterized by scanning electron microscopy (SEM). The presence and stability of key phenolic compounds in propolis were analyzed by high-performance liquid chromatography (HPLC). Optical properties and UV-shielding performance of the coated textiles were evaluated using UV–Vis spectroscopy. The coffee-ring effect was investigated during coating formulation, as this phenomenon significantly reveals coating homogeneity, surface microstructure, and the spatial distribution of active compounds. The influence of formulation parameters on deposit morphology and coating quality was systematically assessed. The biocompatibility of the developed coatings was evaluated through in vitro cytotoxicity testing, demonstrating their suitability for direct skin contact. The combined analytical results confirmed successful incorporation of propolis into the biopolymer matrix, indicating that propolis–biopolymer coatings represent a promising approach for the fabrication of functional textiles intended for topical applications, including wound care, skin protection, and antimicrobial textile products.

## 1. Introduction

The growing demand for sustainable, multifunctional, and biocompatible materials has stimulated considerable interest in the development of functional textiles for healthcare, cosmetic, and biomedical applications. Textiles designed for topical use can serve not only as passive substrates but also as active platforms capable of delivering bioactive compounds directly to the skin [1]. Such systems offer significant potential for wound management, skin protection, antimicrobial treatment, and controlled release applications. The close interaction between skin and clothing has driven interest in therapeutic textiles designed to help manage skin disorders linked to microbiota imbalance. Disrupted skin microbiota is associated with conditions such as atopic dermatitis, psoriasis, seborrheic dermatitis, rosacea, acne, and hidradenitis suppurativa, often accompanied by inflammation, itching, and discomfort [2]. While standard treatments like steroids and antibiotics can provide relief, their long-term use may cause adverse effects, disrupt beneficial microbiota, promote antibiotic resistance, and lead to frequent relapses. In this context, therapeutic textiles have emerged as a promising non-pharmacological approach to alleviate symptoms and help control disease severity [2].

The incorporation of naturally derived materials into textile coatings represents an attractive strategy for producing environmentally friendly and biologically active products while reducing dependence on synthetic additives. A common approach to producing bio-functional textiles is embedding active small molecules within a crosslinked polymer network that is subsequently coated onto fabrics. These coatings act as drug reservoirs, enabling controlled, sustained release and extended interaction with the skin [3].

Propolis, a resinous substance collected by honeybees from plant exudates and mixed with wax and enzymes, has been widely recognized and applied on textiles for its diverse biological activities [4,5]. Numerous studies have demonstrated its antimicrobial, antioxidant, anti-inflammatory, antiviral, and wound-healing properties, which are mainly attributed to its rich composition of polyphenols, flavonoids, phenolic acids, and aromatic compounds. Owing to these characteristics, propolis has attracted increasing attention as a natural functional ingredient in pharmaceutical, cosmetic, and biomedical formulations. However, the direct application of propolis to textile substrates is often limited by its complex composition, poor water solubility, and challenges associated with achieving homogeneous distribution and long-term stability on fiber surfaces.

Biopolymers provide a promising solution to these limitations. Key biopolymers such as bacterial and plant-derived nanocellulose, lignin, chitosan, alginate, gelatin, collagen, keratin, and polylactic acid (PLA) are widely used to impart antimicrobial, flame-retardant, UV-protective, and antioxidant functions. Beyond textiles, these sustainable, biodegradable materials serve as eco-friendly alternatives to petroleum-based polymers in applications including tissue engineering, drug delivery, wound healing, and cosmetics [6]. Natural polymers such as chitosan, alginate, gelatin, starch, and cellulose derivatives possess excellent film-forming abilities, biodegradability, and biocompatibility, making them suitable matrices for the immobilization and controlled release of bioactive compounds. Furthermore, biopolymer-based coatings can improve the adhesion of active substances to textile fibers, enhance durability, and provide additional functionalities such as moisture management and mechanical stability [6]. The combination of propolis with biopolymers may therefore generate synergistic effects, resulting in coatings with improved performance for topical applications [4].

The functionality of coated textiles strongly depends on the coating morphology, chemical interactions between the coating components and the substrate, and the spatial distribution of active compounds. During the drying of liquid formulations, complex physicochemical processes occur that can significantly influence the final coating structure. One phenomenon of particular importance is the coffee-ring effect, whereby suspended particles or dissolved compounds migrate toward the edge of an evaporating droplet, producing non-uniform deposits [7]. In coating technologies, this effect may lead to heterogeneous distribution of active ingredients, variations in coating thickness, and reduced functional performance. Understanding and controlling the coffee-ring effect is therefore essential for the fabrication of homogeneous and reproducible bioactive textile coatings.

Despite the extensive literature describing propolis- or biopolymer-based functional coatings, important knowledge gaps remain regarding the relationship between extract composition, coating morphology, and the resulting physicochemical and biological properties of coated textiles. Systematic comparisons of hydroalcoholic and oil-based propolis extracts incorporated into different biopolymer matrices remain scarce, while the influence of these formulations on coating homogeneity and coffee-ring formation has received limited attention. Moreover, only a few studies have attempted to correlate the chemical composition of propolis extracts with coating morphology and cytocompatibility using complementary analytical techniques (for example HPLC and SEM-EDX studies). Addressing these relationships is essential for the rational design of reproducible and effective bioactive textile coatings intended for topical applications.

Comprehensive characterization is required to evaluate the successful incorporation of propolis into biopolymer matrices and to assess the resulting coating properties. Fourier-transform infrared spectroscopy (FTIR) is commonly employed to investigate chemical interactions and confirm the presence of functional groups associated with both propolis and biopolymers [8]. Surface morphology, coating coverage, and microstructural features can be examined using scanning electron microscopy (SEM). High-performance liquid chromatography (HPLC) enables the identification and quantification of characteristic phenolic compounds responsible for the biological activity of propolis, while UV–VIS spectroscopy provides valuable information regarding optical behavior and ultraviolet protection properties [9]. In addition, cytotoxicity assessment is crucial for determining the biocompatibility and safety of coated textiles intended for direct skin contact.

Incorporation of propolis into biopolymer materials can be enhanced by usage of sophisticated technology such is electrospinning [10] or encapsulation [11,12,13], and application of machine learning and mathematical modeling [14,15,16]. The aim of the present study was to develop textile coatings based on propolis and biopolymers for topical applications and to investigate their physicochemical, morphological, and biological properties. Emphasis was placed on understanding the influence of formulation composition on coating formation and the coffee-ring effect. The prepared coatings were characterized using FTIR, SEM, HPLC, and UV–VIS spectroscopy, while their cytocompatibility was evaluated through in vitro cytotoxicity testing [17,18]. The obtained results contribute to the development of sustainable bioactive textile materials with potential applications in wound care, skin-contact therapeutics, and advanced healthcare textiles.

The novelty of this study lies in the systematic comparison of hydroalcoholic and olive oil propolis extracts incorporated into chitosan- and alginate-based coating matrices, with particular emphasis on the influence of extraction medium and biopolymer composition on coating formation and morphology. Furthermore, this work provides an integrated evaluation of the relationship between the chemical composition of propolis extracts (HPLC), coating structure and surface morphology (SEM), molecular interactions (FTIR), optical properties (UV–VIS spectroscopy), and cytocompatibility within a single comprehensive study. The investigation of coffee-ring-related deposition patterns and their influence on coating homogeneity further contributes to a better understanding of the factors governing the formation of bioactive propolis-based textile coatings.

## 2. Results and Discussion

### 2.1. Results of Fourier Transform Infrared Spectroscopy (FTIR)

FTIR spectra of the coated textile samples confirmed the successful incorporation of propolis into the biopolymer matrix. Characteristic absorption bands corresponding to hydroxyl groups (3200–3500 cm^−1^), aliphatic C–H stretching vibrations (2800–3000 cm^−1^), carbonyl groups (approximately 1700 cm^−1^), and aromatic ring vibrations associated with flavonoids and phenolic compounds were observed. The results of samples with and without modifications are shown in Table 1, Table 2, Table 3 and Table 4.

FTIR analysis was performed to investigate the chemical composition of the prepared propolis-based biopolymer coatings and to identify possible interactions between the textile coating components and the incorporated propolis constituents. The spectra of the prepared coatings exhibited characteristic absorption bands originating from both the biopolymer matrices and bioactive compounds present in propolis, confirming the successful incorporation of propolis into the coating systems.

In alginate-based coatings, the broad absorption region observed between approximately 3200 and 3500 cm^−1^ was assigned to overlapping O–H stretching vibrations originating from hydroxyl groups present in alginate and phenolic compounds of propolis. Due to the broad nature of this region and the possible contribution of residual moisture, this band was not considered as independent evidence of specific interactions. However, differences in band shape and intensity compared with the spectra of the individual components indicate changes in the local hydrogen-bonding environment after incorporation of propolis. The bands observed in the region of approximately 1590–1610 cm^−1^ and 1400–1420 cm^−1^ correspond to asymmetric and symmetric stretching vibrations of alginate carboxylate groups, respectively. Changes in their relative intensity and slight variations in position after addition of propolis suggest interactions between alginate functional groups and oxygen-containing groups of propolis constituents, including phenolic compounds. Furthermore, changes in the fingerprint region (approximately 1000–1150 cm^−1^), associated mainly with C–O–C and C–O stretching vibrations of the polysaccharide backbone, indicate the contribution of overlapping vibrations from both alginate and propolis-derived compounds.

The FTIR spectra of chitosan–propolis coatings showed characteristic absorption bands associated with the polysaccharide structure and functional groups of chitosan together with signals originating from propolis components. A broad absorption band in the range of approximately 3200–3500 cm^−1^ was attributed to overlapping O–H and N–H stretching vibrations. Although this region can also be affected by residual water or solvent-related contributions, changes in band profile and intensity compared with the spectra of the individual components suggest modifications in the hydrogen-bonding environment within the coating matrix. The characteristic amide I and amide II bands of chitosan, located approximately in the regions of 1630–1650 cm^−1^ and 1540–1560 cm^−1^, respectively, showed changes after propolis incorporation, indicating possible interactions involving amino groups of chitosan and oxygen-containing functional groups of propolis constituents. In addition, variations in the C–H stretching region (approximately 2870–2930 cm^−1^) and in the fingerprint region (approximately 1000–1150 cm^−1^) were observed, reflecting contributions from both the chitosan backbone and propolis-derived aromatic and ether-containing compounds.

A broad absorption band in the region of ~3200–3500 cm^−1^ was observed, which can be attributed to overlapping O–H and N–H stretching vibrations originating from hydroxyl groups of the biopolymer matrix and phenolic compounds of propolis. Since this spectral region is highly sensitive to intermolecular hydrogen bonding and may also include contributions from residual moisture or solvent molecules, it was not considered as standalone evidence of specific interactions. However, changes in band shape, position, and intensity compared with the spectra of the individual components suggest alterations in the hydrogen-bonding environment, which may result from interactions between chitosan amino/hydroxyl groups and hydroxyl groups of propolis constituents. These findings were therefore interpreted as complementary evidence and considered together with changes observed in other characteristic FTIR absorption bands.

When comparing alginate- and chitosan-based coatings, the spectral changes observed after propolis incorporation indicate that the type of biopolymer matrix influences the interaction environment of the incorporated bioactive compounds. Chitosan provides additional amino functionalities that can participate in hydrogen bonding and ionic interactions, whereas alginate interactions are primarily associated with hydroxyl and carboxylate groups. However, FTIR analysis alone cannot conclusively determine the exact nature or strength of these interactions; therefore, the observed spectral changes are interpreted as complementary evidence of interactions between propolis constituents and the biopolymer matrices.

Therefore, the FTIR results confirm the presence of characteristic functional groups from both propolis and the selected biopolymers and demonstrate that incorporation of propolis modifies the chemical environment of the coating matrices. These findings, together with SEM observations and HPLC analysis, support the successful formation of propolis-containing functional coatings on textile substrates.

The effect of chitosan and alginate on standard propolis extracts was examined and the results are presented in Table 5. 

Compared with the untreated textile substrate, coated samples exhibited increased absorbance in regions characteristic of propolis-derived phenolic compounds. Slight shifts in selected absorption bands suggested the formation of intermolecular interactions, including hydrogen bonding between the biopolymer matrix and propolis constituents. 

When comparing all obtained FTIR spectroscopy results, those confirm that the successful incorporation of propolis into both chitosan- and alginate-based coating systems was achieved. This is evidenced by the presence of characteristic absorption bands originating from the biopolymer matrices together with functional groups of propolis constituents. In all formulations, the broad absorption band at approximately 3200–3500 cm^−1^ was assigned to overlapping O–H and, in the case of chitosan, N–H stretching vibrations. Changes in shape and intensity relative to the individual components indicate modifications of the hydrogen-bonding environment after propolis incorporation, although this region alone cannot be considered definitive evidence of specific interactions because it is also influenced by residual moisture.

The main differences between the two biopolymer matrices were observed in the spectral regions corresponding to their characteristic functional groups. Chitosan-based coatings exhibited changes in the amide I (≈1630–1650 cm^−1^) and amide II (≈1540–1560 cm^−1^) bands, suggesting interactions between chitosan amino groups and oxygen-containing constituents of propolis. In contrast, alginate-based coatings showed more pronounced variations in the asymmetric and symmetric stretching vibrations of carboxylate groups (≈1590–1610 and 1400–1420 cm^−1^), indicating that interactions were governed primarily by hydroxyl and carboxylate functionalities.

Comparison of the two propolis extracts further revealed differences in spectral intensity rather than the appearance of new absorption bands. Coatings prepared with the hydroalcoholic extract exhibited more pronounced changes in regions associated with phenolic compounds, reflecting the higher extraction efficiency of polar bioactive constituents. In contrast, coatings containing the olive oil extract showed relatively weaker changes in these regions together with a greater contribution of aliphatic C–H stretching vibrations originating from lipid components. In all formulations, modifications within the fingerprint region (approximately 1000–1150 cm^−1^) confirmed the contribution of both the polysaccharide backbone and propolis-derived compounds.

From those results it is evident that the type of propolis extract and the biopolymer matrix influence the chemical environment within the coating, while all formulations exhibited spectral changes consistent with successful incorporation of propolis into the coating layer. These findings indicate successful immobilization of bioactive compounds within the coating layer.

### 2.2. Results of Scanning Electron Microscopy (SEM)

A reference single-component fabric made of 100% cotton fibers was used in this study, supplied by the authorized manufacturer of textile testing reference materials, SDC Enterprises Limited, UK. According to the manufacturer, the fabric is intended for testing the color-fastness of textile materials to various influences and is produced in accordance with the specifications defined in the latest edition of the HRN ISO 105-F standard series [19]. 

Samples of textile carriers were investigated before and after coating with propolis and its mixtures with chitosan and alginate, and results are presented in Table 6 and Table 7. 

Although the SEM evaluation was primarily qualitative, the observed morphological differences are consistent with the chemical composition of the corresponding propolis extracts determined by HPLC. The hydroalcoholic extract, which contained a substantially higher concentration of galangin than the oil-based extract, produced a more homogeneous and continuous coating on the textile fibers. In contrast, the lower flavonoid content of the olive oil extract was associated with localized deposits and less uniform surface coverage. Besides the concentration of extracted bioactive compounds, the different physicochemical properties of the extraction media, including polarity, viscosity, and wetting behavior, are also expected to influence coating formation during drying. These observations suggest that both extract composition and solvent characteristics contribute to the final coating morphology. While SEM successfully confirmed coating deposition and enabled comparison between formulations, quantitative evaluation of coating coverage and surface roughness was beyond the scope of the present study and will be addressed in future work.

SEM micrographs confirmed the presence of coating deposits on the textile fibers following treatment with the different propolis formulations. The untreated textile exhibited a relatively smooth fiber surface, whereas coated samples showed continuous surface deposits and coating films.

Samples prepared from hydroalcoholic formulations appeared to exhibit a more continuous and evenly distributed coating along the fiber surface than those prepared from the oil-based extract, which showed more localized deposits and a less uniform apparent surface morphology. These observations are based solely on qualitative visual assessment of the SEM micrographs and should not be interpreted as a quantitative evaluation of coating homogeneity.

The observed qualitative morphological differences are consistent with the chemical composition of the corresponding propolis extracts determined by HPLC. The hydroalcoholic extract, which contained a substantially higher concentration of galangin than the oil-based extract, appeared to produce a more continuous surface coating, whereas the lower flavonoid content of the olive oil extract was associated with more localized deposits. In addition to differences in the concentration of extracted bioactive compounds, the physicochemical properties of the extraction media, including polarity, viscosity, and wetting behavior, are also expected to influence coating formation during solvent evaporation and drying. These factors likely contribute to the observed differences in coating morphology.

It should be emphasized that the SEM analysis performed in this study is qualitative and serves to illustrate the apparent morphological differences between the investigated formulations. Quantitative determination of coating coverage, coating homogeneity, surface roughness, or coating thickness was beyond the scope of the present work. Therefore, the SEM observations should be interpreted as qualitative evidence of coating deposition rather than quantitative proof of coating uniformity. Future studies incorporating quantitative image analysis and complementary surface characterization techniques will be required to objectively evaluate coating homogeneity and surface coverage.

### 2.3. High-Performance Liquid Chromatography (HPLC) Analysis 

**Figure 1:**
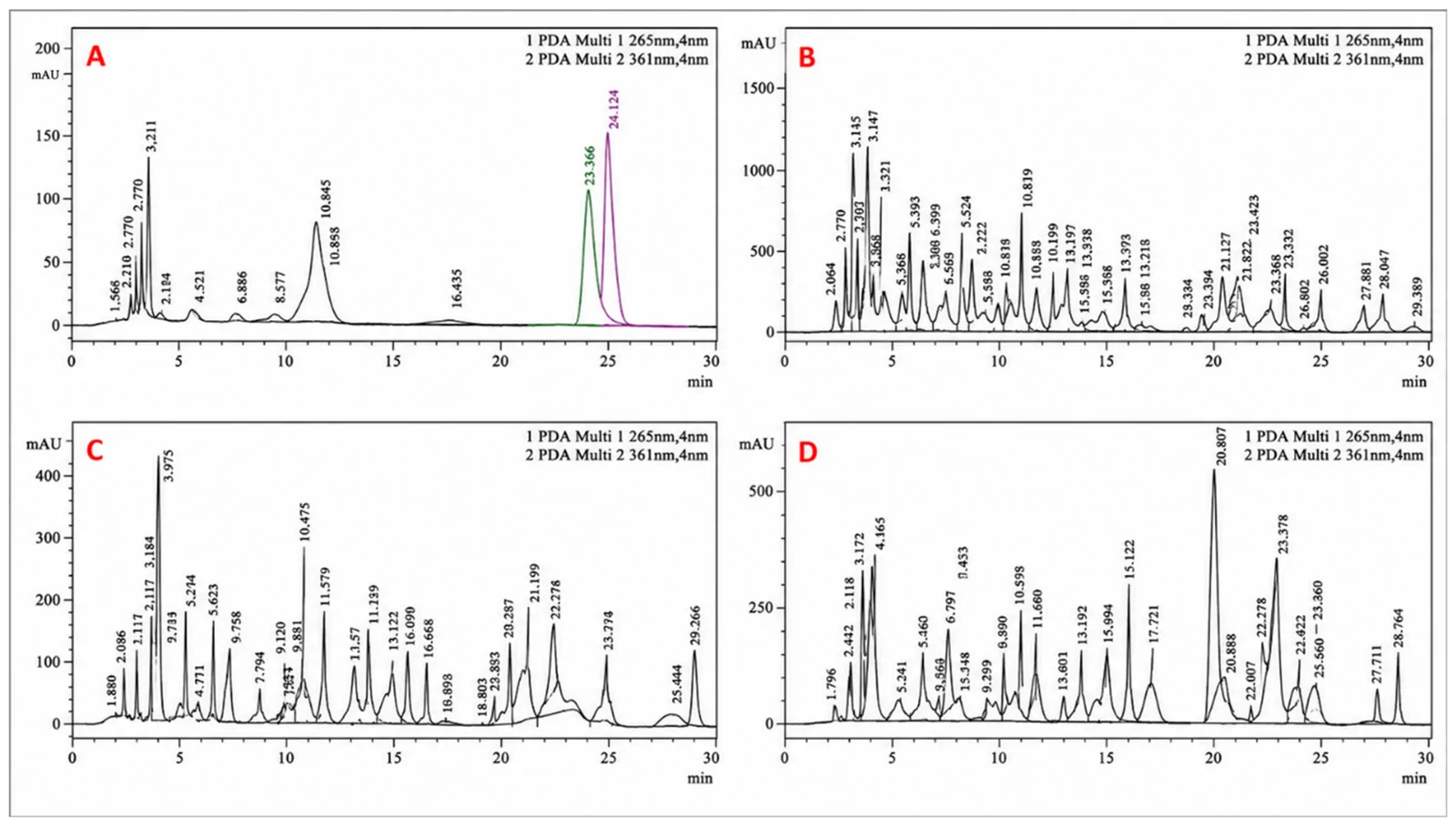
HPLC chromatograms with data on retention time, area and height of the peak for galangin in investigated samples: (**A**) standard galangin solution at 265 nm (pink line) and 361 nm (green line), (**B**) the hydroalcoholic extract (Sample 1-1), (**C**) olive oil extract (Sample 2-1), and (**D**) the standard propolis extract (Sample A-1).

HPLC analysis was performed to quantify galangin as a marker flavonoid of propolis. Galangin was selected as a marker compound because it is one of the characteristic flavonoids present in propolis and contributes significantly to its antioxidant and antimicrobial activity. Resulting HPLC chromatograms presented inf Figure 1 enabled the comparison of galangin concentrations among the hydroalcoholic extract (Sample 1-1), olive oil extract (Sample 2-1), and the commercially available standard propolis extract (Sample A-1).

The chromatographic analysis revealed a characteristic galangin peak at a retention time of 23.35 min (at 265 nm) and 23.29 min (at 361 nm). Quantitative analysis demonstrated that Sample 1-1 contained 1449.3 mg/L galangin, while Sample 2-1 contained 248 mg/L. The commercial sample (A-1) exhibited a galangin concentration of 1864 mg/L.

In the present study, galangin was selected as the target compound for HPLC analysis due to its consistent occurrence as one of the characteristic flavonoids present in propolis, independent of geographical origin and botanical source. Galangin has been extensively reported as a representative propolis constituent, and its molecular and crystal structure have been well documented. Owing to its abundance, chemical stability, and biological relevance, galangin is frequently used as a marker compound for evaluating the flavonoid content and extraction efficiency of propolis-based formulations. Therefore, the determination of galangin concentration was used in this study as an indicator of the efficiency of different extraction systems and the successful incorporation of bioactive propolis constituents into the developed textile coatings.

The results confirmed that galangin was successfully detected in all tested samples, with the hydroalcoholic extract (1-1) showing the highest concentration, followed by the commercial extract (A-1), while the oil-based extract (2-1) exhibited the lowest content. The higher galangin concentration observed in Sample 1-1 confirms the superior extraction efficiency of the hydroalcoholic solvent system for flavonoid recovery. Ethanol effectively solubilizes flavonoids and phenolic compounds due to its intermediate polarity, allowing efficient extraction of both moderate polar and non-polar constituents. In contrast, the olive oil extract contained lower concentrations of galangin, which is consistent with previously reported lower extraction efficiencies of oil-based systems. Nevertheless, the presence of detectable galangin in Sample 2-1 demonstrates that olive oil can successfully extract biologically relevant flavonoids and may therefore represent a suitable alternative extraction medium for applications where alcohol-free formulations are desired.

The results obtained confirm that ethanol–water mixtures provide significantly higher extraction efficiency for flavonoids compared to oil-based systems. 

This is consistent with the higher polarity of hydroalcoholic solvents, which enables efficient solubilization of phenolic compounds such as galangin. Despite lower efficiency, the presence of galangin in the oil-based extract confirms that olive oil can still extract biologically relevant flavonoids, supporting its potential use in alcohol-free topical formulations.

The HPLC analysis performed in this study was used primarily for comparative evaluation of the propolis extracts and confirmation of differences in galangin content between the investigated extraction systems. Although the obtained results demonstrate differences in extraction efficiency between hydroalcoholic and oil-based formulations, a complete analytical validation of the method, including linearity, accuracy, precision, recovery, and comprehensive statistical evaluation, was beyond the scope of the present work. Future studies will include full validation of the chromatographic method and statistical assessment based on replicate measurements to provide more detailed quantitative information regarding propolis composition.

The differences observed in the SEM micrographs are consistent with the HPLC results. Quantitative chromatographic analysis revealed that the hydroalcoholic extract (Sample 1-1) contained a considerably higher concentration of galangin (1449.3 mg/L) than the olive oil extract (Sample 2-1, 248 mg/L), confirming the superior extraction efficiency of the ethanol–water solvent system for flavonoids and other phenolic compounds. This higher concentration of bioactive constituents is reflected in the coating morphology, as the hydroalcoholic formulation produced a more homogeneous and continuous coating along the textile fibers. In contrast, the oil-based extract, containing a substantially lower galangin concentration, resulted in localized accumulations and a less uniform surface morphology. Although coating formation is also influenced by the physicochemical properties of the extraction medium, the results suggest that the greater content of extractable phenolic compounds in the hydroalcoholic formulation contributed to more uniform deposition on the fiber surface. Thus, the HPLC and SEM analyses provide complementary evidence that the extraction solvent influences both the chemical composition of the extract and the morphology of the resulting functional coating.

### 2.4. Results of UV–Visible Spectroscopy

UV–Visible spectroscopy Lambda 20, Perkin Elmer Corporation, MA, USA, was used to evaluate the optical properties of the prepared extracts and coated textile samples. Absorbance measurements were performed within the ultraviolet and visible spectral regions to assess the presence of UV-absorbing compounds and the potential ultraviolet protective properties imparted by the propolis-containing coatings. 

**Figure 2:**
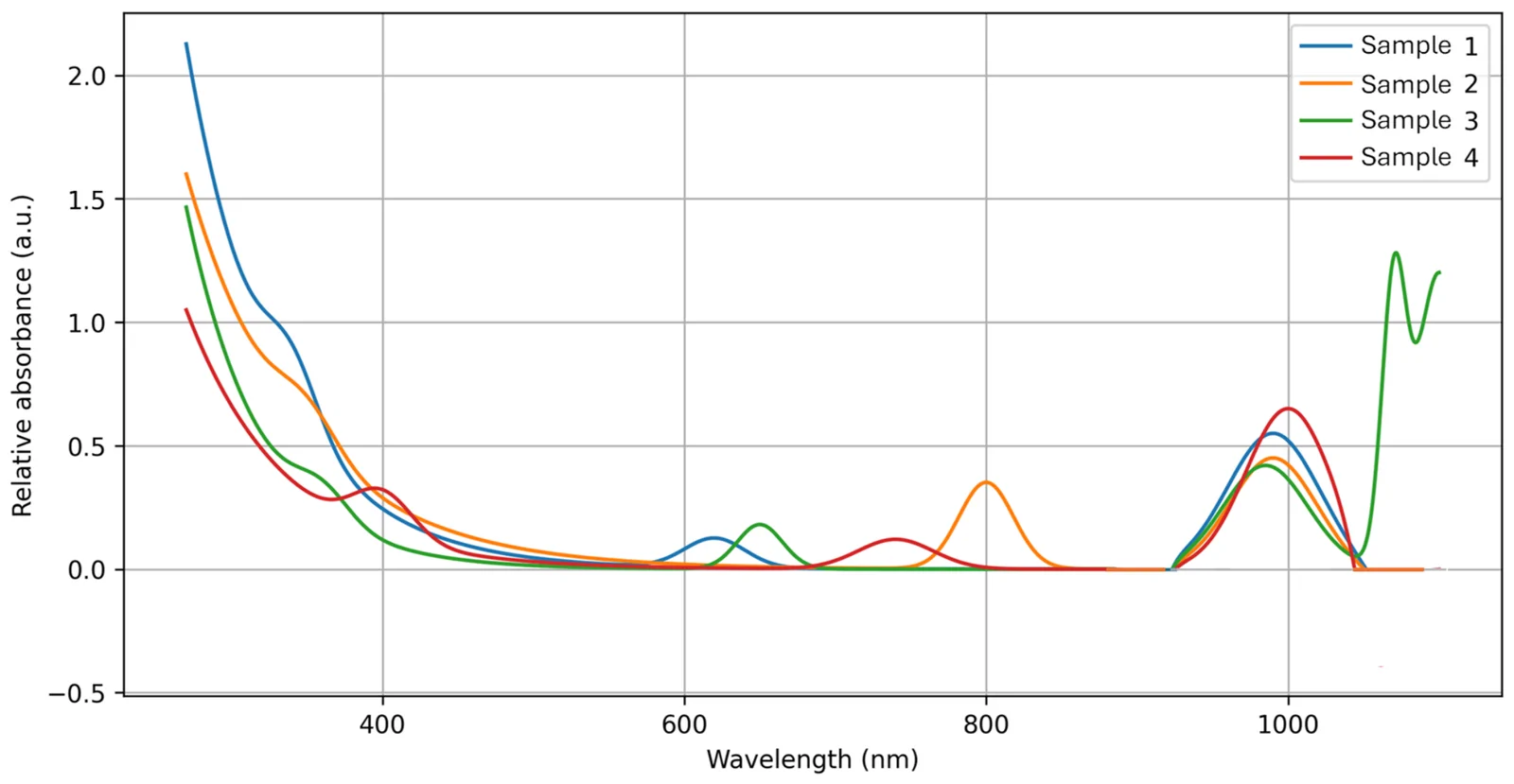
Obtained spectra recorded in UV-VIS range of 200 to 1100 nm, for samples: (1) blue line corresponds to sample 1, pure propolis extract; (2) orange line to sample 2, propolis extract with olive oil component in emulsion; (3) green line to propolis sample with chitosan; (4) red line to propolis sample with alginate.

**Figure 3:**
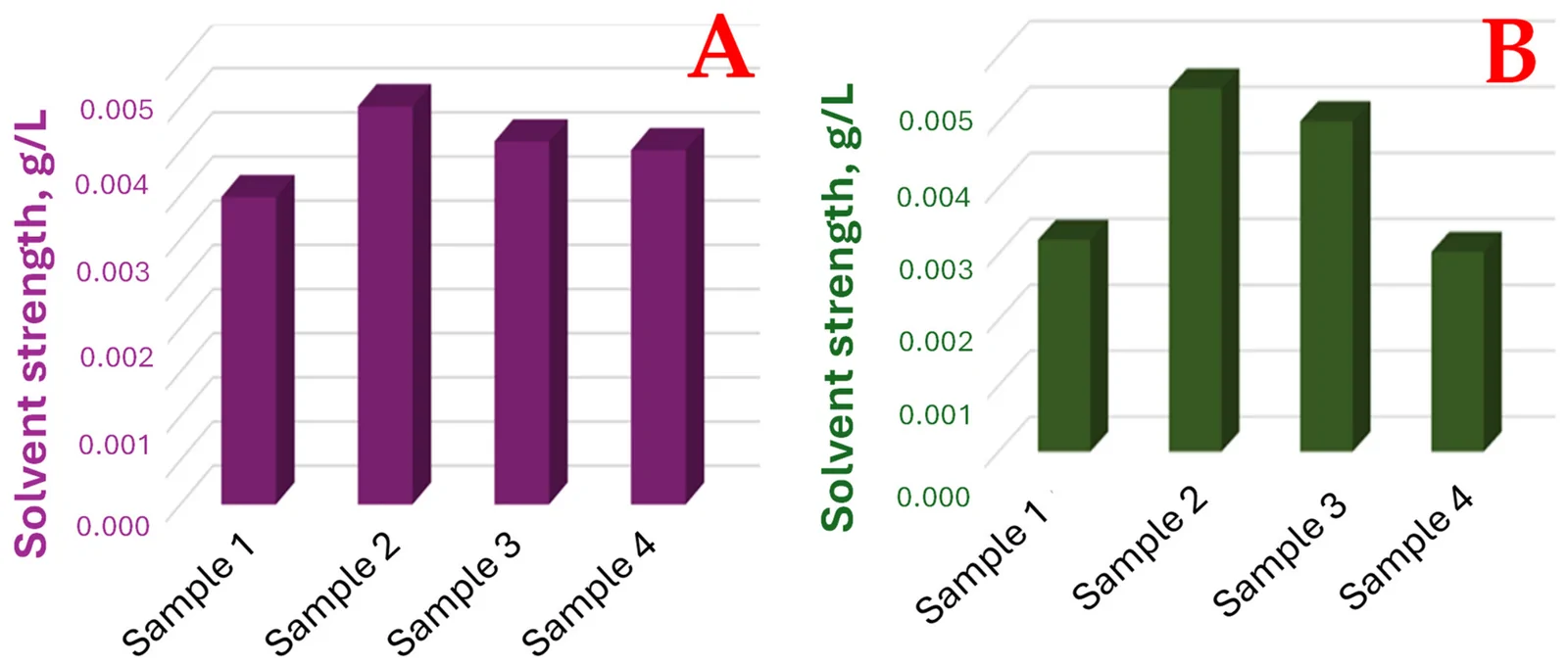
Relative comparison of solvent strength in preparation of propolis extracts for application on textile coatings foreseen in topical applications (**A**) at a wavelength of 984.24 nm and (**B**) 350.45 nm.

UV–VIS spectroscopy demonstrated significant absorption in the ultraviolet region for both propolis extracts and mixtures prepared for coating of textile samples. The absorption bands are attributable to flavonoids and phenolic acids naturally present in propolis (Figure 2 and Figure 3).

As can be seen from the UV-VIS results, samples show activity in the UV region, so the coated textiles can exhibit enhanced UV-blocking properties compared with untreated textile substrates. Samples prepared with the hydroalcoholic extract generally showed stronger UV absorption due to the higher concentration of extracted phenolic compounds, so propolis-containing coatings may contribute to improved UV protection in addition to their biological functionality.

UV–VIS spectroscopy was used to evaluate the optical absorption properties of the propolis-based coatings and to confirm the presence of UV-absorbing compounds originating from propolis constituents. The observed absorption behavior can be attributed mainly to phenolic compounds and flavonoids, which contain conjugated structures capable of absorbing radiation in the UV region. Differences in the UV–Vis spectra between the investigated formulations reflect variations in the composition and concentration of extracted bioactive compounds, consistent with the HPLC results.

Although increased UV absorption of the coating formulations indicates potential UV-screening capability, UV–VIS measurements alone cannot be directly correlated with the UV-protective performance of textile materials. The ultraviolet protection factor (UPF) is a textile-specific parameter that depends not only on the intrinsic absorption properties of the coating but also on factors such as coating thickness, fiber structure, porosity, coverage uniformity, and durability during use. Therefore, standardized measurements of UV transmittance and UPF according to relevant textile testing procedures are required to confirm the protective performance of the developed materials. The present UV–VIS results should therefore be considered as an indication of the UV-absorbing potential of propolis-based coatings, while comprehensive UPF evaluation and durability testing will be addressed in future investigations.

UV-VIS investigation also revealed the solvent strengths of the applied solvents, as is shown in Figure 3.

Figure 3 presents a qualitative comparison of the extraction efficiencies of the investigated solvents. This experiment was designed as an initial screening to evaluate the relative extraction performance of different solvent systems and to identify the most suitable extraction medium for further analyses. As the objective was comparative rather than quantitative, replicate experiments were not performed and, consequently, no statistical analysis was applied. The results should therefore be interpreted as indicative of the relative extraction efficiency of the investigated solvent systems.

Comparison of the two extraction systems demonstrated that solvent selection significantly influences the chemical composition of propolis extracts and consequently the properties of the resulting coatings. The hydroalcoholic extraction system produced higher concentrations of galangin and other phenolic compounds, resulting in stronger UV absorption and more pronounced bioactive functionality. Conversely, the olive oil extraction system yielded lower flavonoid concentrations but provided an alcohol-free alternative capable of retaining selected lipophilic bioactive constituents. Each extraction approaches successfully generated functional coatings, although differences in coating morphology and active compound distribution were observed.

### 2.5. Results of the Coffee-Ring Effect

The drying process produced visible manifestations of the coffee-ring effect. 

**Figure 4:**
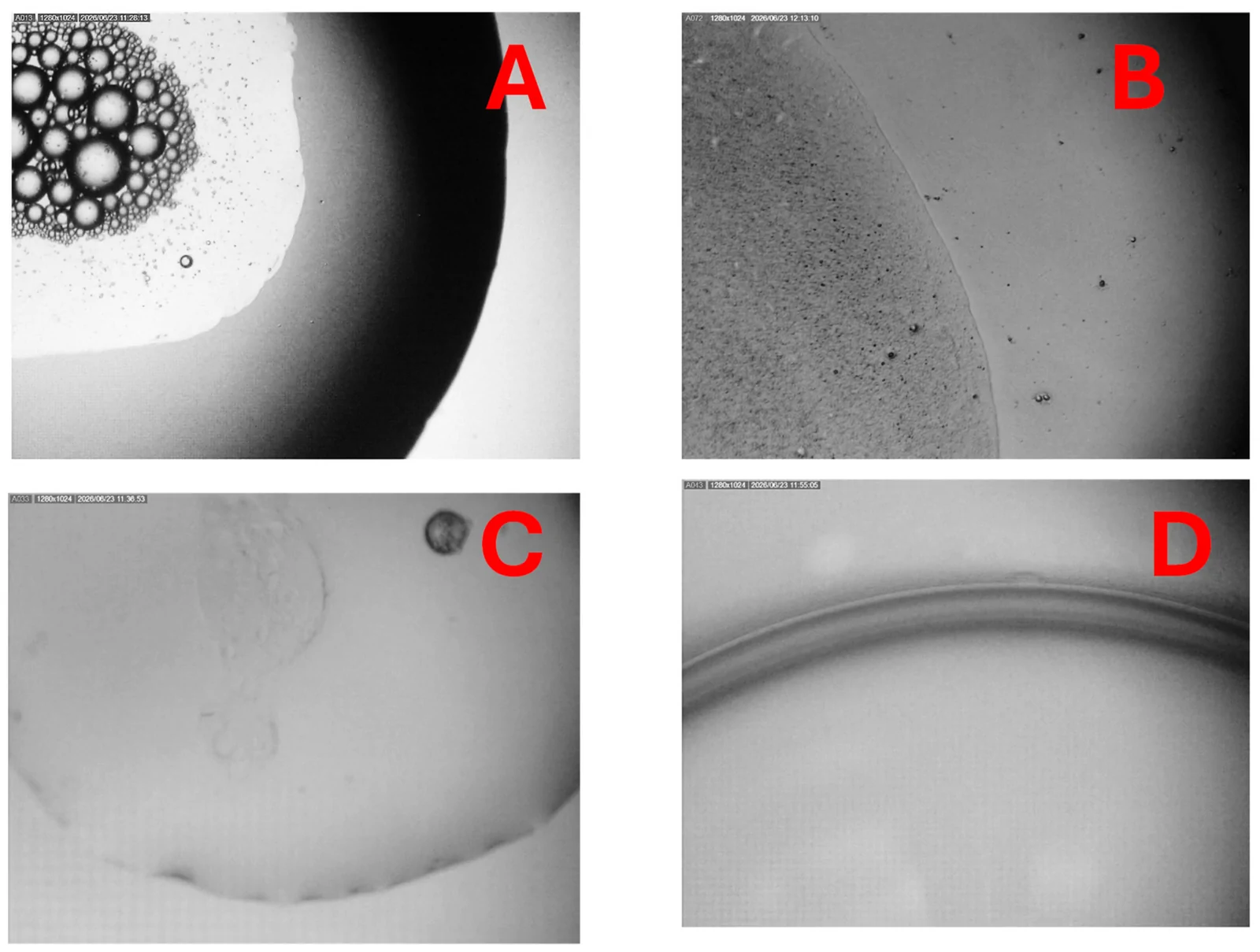
Coffee ring effects recorded under digital microscope DinoLite, for samples: (**A**) sample 1, distinctive oil components preserved; (**B**) sample 2, no oil components present in the sample, ring not distinguished; (**C**) sample 3, smaller oil components; (**D**) sample 4, two rings obtained completely separated.

The coffee-ring effect was evaluated by analyzing the morphology and spatial distribution of deposited material following drying of coating droplets (Figure 4). Optical observations and Dino-Light digital microscope (Dunwell Tech, Inc., Los Angeles, CA, USA) imaging were used to assess the formation of peripheral deposits and coating heterogeneity. The influence of extraction medium and formulation composition on deposit morphology was compared to determine factors contributing to more uniform coating formation.

The observed reduction in coffee-ring formation is advantageous for topical applications because homogeneous coatings are expected to provide more reproducible biological activity and controlled release behavior. Peripheral accumulation of coating material was observed in some deposited droplets, indicating capillary-driven transport of suspended and dissolved constituents during solvent evaporation. However, the presence of biopolymers appeared to reduce particle migration and contributed to a more uniform distribution of active compounds.

The formation of non-uniform deposits observed in some coated samples can be associated with the coffee-ring effect, which occurs during evaporation-driven drying of liquid droplets. In this phenomenon, capillary flow within the evaporating droplet promotes migration of dissolved compounds or suspended particles toward the droplet perimeter, resulting in localized accumulation of deposited material. In the present study, the coffee-ring effect was evaluated qualitatively based on SEM observations, where differences in coating distribution and surface morphology were observed between formulations. Hydroalcoholic propolis formulations resulted in more homogeneous surface coverage, whereas oil-based formulations showed more pronounced localized deposits. These differences may arise from variations in extract composition and physicochemical properties of the coating solutions, including polarity, viscosity, and evaporation behavior.

Although quantitative parameters describing coffee-ring intensity, coating thickness distribution, or spatial deposition gradients were not determined in the present study, the observed morphological differences demonstrate the importance of controlling drying and deposition processes in the development of reproducible bioactive textile coatings. Future studies will include quantitative image analysis and surface mapping approaches to evaluate the coffee-ring effect and its influence on coating performance.

### 2.6. Cytotoxicity Testing

The biocompatibility of the coated textile samples was evaluated by in vitro cytotoxicity testing included untreated cells as a negative control representing 100% cell viability and DMSO-treated cells as a positive cytotoxic control to confirm the sensitivity of the assay. Human keratinocytes (HaCat, were generous gift from colleague from the University Zagreb) were cultured in Dulbecco’s Modified Eagle’s Medium (DMEM) supplemented with 10% (*v*/*v*) foetal bovine serum (FBS), and 1% (*v*/*v*) penicillin/streptomycin (PenStrep solution). Measurements were performed using independent experimental replicates, and the obtained viability values were expressed relative to untreated control. 

**Figure 5:**
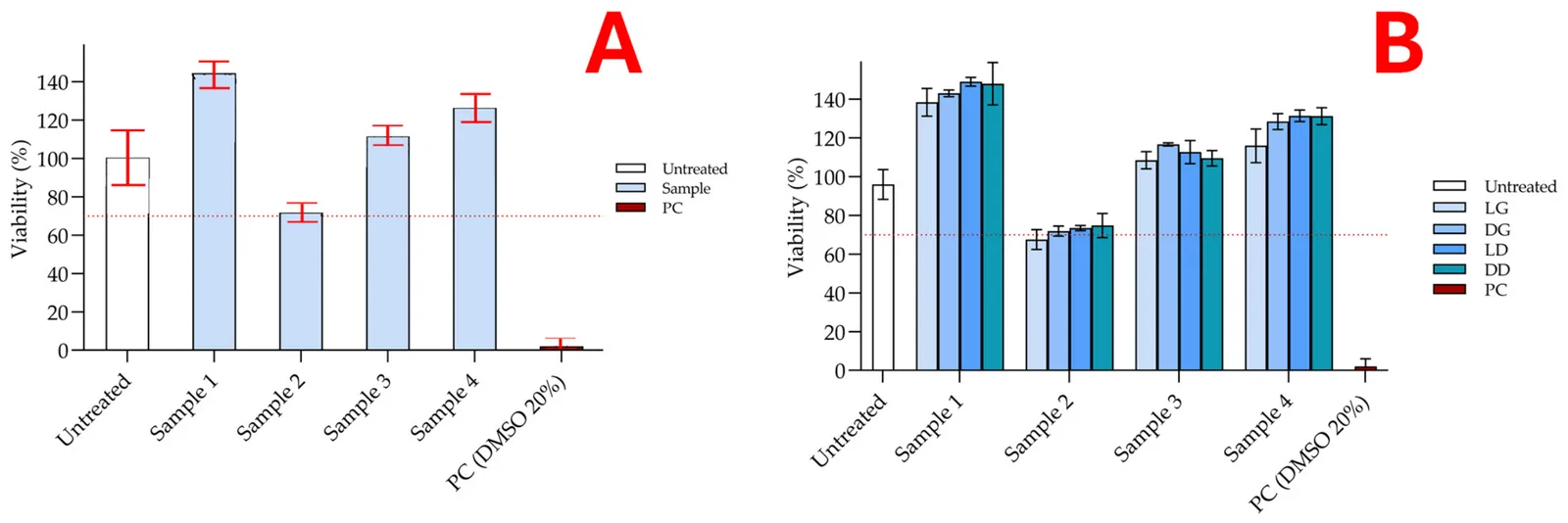
Viability of HaCat cells exposed to textile samples (1–4) after 7-day extraction in cell culture medium supplemented with fetal bovine serum (FBS) for 24 h. (**A**) Pooled data representing all four quadrants of each textile sample (1–4) and (**B**) separately for each individual quadrant: upper left (LG), lower left (LD), upper right (DG), and lower right (DD). Untreated cells represent cells treated only with cell medium and 20% DMSO was used as positive control (PC). Red dotted line represents 70% threshold representing ISO 10993-5 standard.

The results of the MTS assay on HaCaT cells after 24 h exposure to extracts released from the samples (obtained by immersing samples in cell culture medium supplemented with fetal bovine serum (FBS) for 7 days prior to experiments), are presented in Figure 5. Compared with untreated control, Samples 1, 3, and 4 had no negative influence on cell viability, indicating preserved cellular metabolic activity. Moreover, it seems that samples 1 and 4 enhanced metabolic activity relative to the control. Only the sample 2 lowered viability compared with both the control and the other samples tested; however, the measured value remained above 70% in the tested time frame. The positive control (20% DMSO) resulted in almost complete loss of cell viability, confirming the sensitivity and validity of the applied method.

The cytotoxicity evaluation demonstrated that the prepared coatings were compatible with the tested cell line under the experimental conditions employed. Cell viability values exceeded the threshold commonly accepted for non-cytotoxic materials (70%), indicating good biocompatibility (Figure 5) and complying with the ISO 10993-5 standard [20].

Since all samples maintained HaCaT cell viability above 70%, differences between the formulations can be interpreted primarily in terms of their influence on cellular metabolic activity rather than cytotoxicity. When comparing formulations prepared with different propolis extracts, samples containing the oil-based propolis extract (Samples 1 and 3) positively influenced cell viability/metabolic activity. This extract consisted of propolis dispersed in olive oil with the addition of lavender essential oil, resulting primarily in the extraction of lipophilic propolis constituents. 

Lavender essential oil was incorporated into the olive oil-based propolis extract primarily to improve the sensory properties of the formulation by masking the characteristic odor of propolis. Although lavender essential oil has been reported to possess antimicrobial activity, its concentration in the present study was selected mainly for fragrance and was not intended to serve as the principal antimicrobial agent. The primary objective of this work was to develop and characterize propolis–biopolymer textile coatings and to verify their cytocompatibility, which represents a prerequisite for their intended skin-contact applications. Accordingly, evaluation of the antimicrobial activity of the coated textiles was beyond the scope of the present study. Future investigations will focus on assessing the antibacterial efficacy of the developed coatings against clinically relevant wound pathogens, together with additional biological performance studies to further establish their suitability for wound-care applications.

In contrast, the hydroalcoholic propolis extract (Sample I) contained a substantially higher amount of propolis (87.5 g) extracted in an ethanol–water mixture, enabling the solubilization of a broader range of bioactive compounds, including phenolic acids and flavonoids responsible for many of the biological properties of propolis. Nevertheless, Sample 2, which contained alginate and the hydroalcoholic propolis extract, exhibited the highest influence on the viability among all tested formulations. Since Sample 4 also contained the same propolis extract but did not show reduced viability, the observed decrease in metabolic activity cannot be attributed solely to the composition of the propolis extract or to the use of ethanol during extraction.

Interestingly, the main difference between Samples 2 and 4 was the type of polysaccharide matrix. Sample 2 was based on alginate, whereas Sample 4 contained chitosan dissolved in 0.5% acetic acid. As Sample 4 displayed one of the highest viability values, it can be assumed that chitosan contributed to the preservation of HaCaT cell metabolic activity. This observation is consistent with numerous studies describing chitosan as a highly cytocompatible biopolymer with favorable properties for wound-healing and tissue-engineering applications.

Obtained results suggest that the type of biopolymer has a greater influence on HaCaT cell metabolic activity than the type of propolis extract itself. Chitosan-based formulations (Samples 3 and 4) consistently maintained or enhanced cellular metabolic activity regardless of the extract used, whereas the alginate formulation containing the hydroalcoholic propolis extract (Sample 2) exhibited reduced viability. However, as already mentioned, as cell viability remained above 70% in all cases, none of the tested samples can be considered cytotoxic according to the criteria of ISO 10993-5.

Propolis is a complex natural product rich in phenolic compounds and flavonoids, whose composition and biological activity strongly depend on extraction conditions, geographical origin, and botanical sources. Usman et al. demonstrated that solvent polarity significantly affects the phytochemical profile and antioxidant activity of propolis extracts, with ethanolic extracts generally exhibiting higher phenolic content and antioxidant capacity than aqueous extracts [21]. Similarly, Kurek-Górecka et al. showed that the antioxidant activity of propolis polyphenols depends on their concentration and molecular structure, while synergistic interactions among different phenolic constituents contribute to the overall biological activity of propolis [22]. Comparative studies of aqueous, polyethylene glycol–aqueous, and ethanolic extracts further confirmed that extraction conditions influence the recovery of bioactive compounds, antioxidant properties, and biological responses, highlighting the importance of selecting suitable extraction systems for biomedical applications [23,24,25]. Moreover, concentration and purification approaches, such as nanofiltration, can enhance flavonoid and phenolic content while preserving biological activity, improving the functional potential of propolis-based formulations [24].

The increasing demand for bioactive and sustainable materials has stimulated the development of functional coatings incorporating natural compounds and advanced nanomaterials. In this context, naturally derived antimicrobial agents and functional surfaces represent promising approaches for healthcare applications, particularly considering the growing challenge of antimicrobial resistance. Vukoja et al. demonstrated the antibacterial potential of colloidal platinum nanoparticles against ESBL-producing bacterial strains, illustrating the potential of advanced functional materials as alternatives or complements to conventional antimicrobial strategies [26]. Engineered nanoparticles and biodegradable polymer systems have been investigated for imparting antimicrobial, protective, and catalytic properties to textile materials, while green synthesis strategies and sustainable processing approaches provide environmentally responsible alternatives for biomedical applications [27,28,29].

The biological activity of propolis is primarily associated with its diverse phenolic composition, including flavonoids, phenolic acids, and related compounds. Their antioxidant activity is governed by structural characteristics such as hydroxyl group arrangement, electron-donating ability, and capacity to stabilize reactive oxygen species [30,31,32]. Consequently, detailed chemical characterization is essential for understanding the relationship between propolis composition and biological performance. Propolis has been reported to exhibit antioxidant, antimicrobial, anti-inflammatory, immunomodulatory, and wound-healing effects, supporting its potential use in topical formulations and biomedical materials [33,34].

Among propolis constituents, galangin has attracted considerable attention due to its antioxidant, anti-inflammatory, antiproliferative, and cytoprotective activities. Previous studies have demonstrated that galangin can modulate oxidative stress-related pathways, influence apoptosis mechanisms, and protect human keratinocytes against ultraviolet-induced oxidative damage, indicating its relevance for skin-contact applications [35,36,37,38,39]. Although the biological activity of propolis results from the combined action of multiple compounds, galangin is considered one of the important contributors to its radical-scavenging capacity [37,38]. Experimental and computational investigations, including density functional theory (DFT) studies, have further clarified the relationship between molecular structure and antioxidant efficiency of flavonoids and phenolic compounds [39,40,41,42,43,44].

The antioxidant potential of propolis flavonoids is closely related to their chemical structure, including hydroxylation pattern, molecular conjugation, and electron-transfer properties [45]. Galangin has been successfully quantified using chromatographic methods such as HPLC, enabling correlations between chemical composition and biological activity [46,47]. However, propolis composition is highly variable and depends on geographical origin, plant sources, and extraction methodology. Modern analytical approaches, including FTIR, GC-MS, UHPLC-DAD-QqTOF-MS, and LC-MS-based chemometric profiling, have revealed significant chemical diversity among propolis samples and confirmed the importance of comprehensive characterization for establishing relationships between composition and functional properties [48,49].

Phenolic compounds may exhibit both antioxidant and, under specific conditions, prooxidant effects depending on concentration, molecular structure, and biological environment, emphasizing the need for careful evaluation when developing propolis-based biomaterials [50]. Studies of natural bioactive compounds have further demonstrated their potential to reduce oxidative stress and protect biological systems, supporting their application in biomedical and topical formulations [51]. Therefore, combining chemical, morphological, and biological characterization is essential for the rational design of reproducible propolis-containing coatings with controlled properties for textile and healthcare applications [52,53].

Propolis compositional differences are particularly relevant for the development of bioactive textile coatings, as the concentration and chemical nature of extracted compounds directly influence coating functionality, stability, and biological performance.

The biological activity of propolis is primarily associated with its high content of phenolic compounds and flavonoids, which contribute to antioxidant, antimicrobial, anti-inflammatory, and protective effects. Previous investigations have demonstrated that propolis extracts from different geographical origins exhibit broad-spectrum antimicrobial activity, which has been attributed to specific phenolic constituents and their synergistic interactions within the complex extract matrix [54,55,56,57,58,59]. Similarly, ethanolic propolis extracts have shown significant biological activity and cytotoxic effects against selected cancer cell models, demonstrating the importance of extraction conditions in determining the concentration and activity of recovered compounds [55]. The antioxidant capacity of propolis is closely related to the ability of its polyphenolic components to neutralize reactive oxygen species and modulate cellular redox balance. However, depending on concentration and cellular environment, polyphenols may also exhibit pro-oxidant behavior through the formation of phenoxyl radicals, highlighting the importance of controlled incorporation of these compounds into biomedical materials [50,60,61].

The incorporation of propolis into textile coatings requires careful consideration of both biological effectiveness and biocompatibility [62,63,64]. Several studies have investigated the protective and cytogenetic effects of propolis and its flavonoid constituents, demonstrating that propolis extracts may reduce oxidative damage and protect cellular components under different stress conditions [51,58,65,66,67]. For example, propolis and its polyphenolic compounds have shown protective effects against radiation-induced DNA damage in human lymphocytes, suggesting their potential role as natural antioxidant protectants [58,66,67]. Conversely, some studies have investigated the possible genotoxic or cytotoxic effects of propolis extracts, emphasizing that biological responses depend strongly on extract composition, concentration, exposure conditions, and the specific cellular model used [56,68]. Therefore, evaluation of cytocompatibility is essential when developing propolis-based materials intended for direct contact with biological tissues.

The biological mechanisms associated with propolis are increasingly attributed to the synergistic action of multiple polyphenolic compounds rather than to a single active component. Recent studies have highlighted the molecular pathways involved in the anticancer, antioxidant, and protective effects of propolis, including regulation of oxidative stress, apoptosis-related signaling, inflammatory pathways, and cellular defense mechanisms [62,63,64]. Such multifunctionality supports the application of propolis as a natural active ingredient in advanced biomedical materials. In particular, the combination of antioxidant and antimicrobial properties makes propolis an attractive candidate for functional textiles designed for skin-contact applications, wound care, and protective healthcare materials.

In the context of the present study, the incorporation of propolis extracts into chitosan- and alginate-based textile coatings represents an approach to combine the biological activity of propolis with the film-forming and biocompatible properties of natural polymers. Previous studies have demonstrated that propolis extracts can maintain biological activity when incorporated into different biomaterial systems; however, the final performance depends strongly on the extraction procedure, composition of the extract, polymer matrix, and deposition method [48,55]. The differences observed between hydroalcoholic and oil-based propolis formulations in the present work are therefore consistent with previous reports demonstrating that solvent selection determines the phenolic profile and biological potential of propolis extracts [52,53]. Furthermore, the detected bioactive compounds contribute not only to the antioxidant functionality of the coatings but may also influence their interaction with cells and biological environments, supporting the importance of combining chemical characterization with morphological and cytocompatibility evaluation.

Propolis is a complex natural product containing a broad spectrum of phenolic acids, flavonoids, aromatic compounds, and other bioactive constituents, with its chemical composition depending on botanical origin and geographical location. Nevertheless, flavonoids such as galangin are consistently reported as characteristic constituents of propolis and are considered important contributors to its antioxidant, antimicrobial, and anti-inflammatory properties [32,33]. Previous studies have demonstrated that standardized propolis polyphenol extracts exhibit significant antioxidant activity and biological effects, highlighting the importance of flavonoid-rich fractions in determining the functional properties of propolis preparations [34]. Among these compounds, galangin (3,5,7-trihydroxyflavone) has attracted considerable attention due to its multiple biological activities, including antioxidant, cytoprotective, and antiproliferative effects [35,36]. Its antioxidant potential has been associated with its hydroxylated flavone structure and ability to participate in radical scavenging mechanisms, together with other major propolis constituents such as caffeic acid phenethyl ester [37]. Moreover, galangin has been reported to protect human keratinocytes against UVB-induced oxidative stress, suggesting its potential relevance in skin-contact applications where antioxidant and protective functions are desired [38,39].

The extraction efficiency of galangin and other phenolic compounds is strongly influenced by the polarity and physicochemical properties of the extraction medium, so can be studies through electrochemical methods [40,41,42]. Hydroalcoholic systems are widely applied for propolis extraction because ethanol–water mixtures provide favorable polarity for the solubilization of both moderately polar flavonoids and phenolic acids, resulting in higher recovery of bioactive compounds compared with less polar extraction systems [32,34]. In contrast, oil-based extraction systems generally exhibit lower extraction efficiency for highly polar phenolic constituents; however, they may provide advantages for alcohol-free formulations and improve compatibility with topical applications [43]. The detection of galangin in the oil-based extract in the present study confirms that olive oil is capable of extracting relevant flavonoid compounds, although at lower concentrations compared with the hydroalcoholic system. This observation is consistent with previous studies demonstrating that extraction conditions significantly affect the phenolic fingerprint and antioxidant capacity of propolis-derived preparations [44,45].

The biological relevance of galangin is related not only to its antioxidant activity but also to its ability to interact with cellular pathways involved in oxidative stress regulation and inflammation. Studies have demonstrated that galangin can induce apoptosis and inhibit proliferation in different cancer cell models through mechanisms involving mitochondrial pathways and modulation of signaling pathways such as PI3K/Akt and STAT3 [35,36,43]. Furthermore, theoretical and experimental investigations have shown that the antioxidant behavior of flavonoids is closely related to their molecular structure, including hydroxyl group arrangement, electron delocalization, and proton transfer mechanisms [41,42,44,45]. These structural characteristics contribute to the ability of flavonoids to neutralize reactive oxygen species and may explain their protective effects in biological systems.

Although the present study focused on galangin as a representative compound rather than providing a complete compositional profile of propolis extracts, its quantification enabled comparison of the extraction efficiency of different solvent systems and provided insight into the relationship between extract composition and coating characteristics. The higher galangin content detected in the hydroalcoholic extract was consistent with the more homogeneous coating morphology observed by SEM, suggesting that increased availability of phenolic constituents may contribute to improved distribution of bioactive compounds within the biopolymer matrix. Conversely, the lower galangin concentration in the olive oil extract did not eliminate its potential functionality, as olive oil itself contains bioactive components with antioxidant properties and has been reported to provide protection against oxidative stress through its phenolic constituents and major lipid components [46]. Therefore, both extraction systems may represent valuable approaches for developing propolis-based functional coatings, with the selection of extraction medium depending on the targeted application, desired bioactive content, and formulation requirements.

The galangin content was determined using a validated high-performance liquid chromatography (HPLC) method according to Wang (2007), originally described in the study “Quantitative Analysis of Galangin from Propolis in Different Areas by HPLC” published in Zhong Yao Cai (Journal of Chinese Medicinal Materials) [47].

Chromatographic separation was performed on a Diamonsil C18 reversed-phase column using a mobile phase consisting of methanol and 4% (*v*/*v*) phosphoric acid (H_3_PO_4_) in a ratio of 65:35 (*v*/*v*). The mobile phase was delivered under isocratic conditions at a flow rate of 1.0 mL min^−1^. Galangin was detected by UV absorbance at 256 nm. Under these chromatographic conditions, the method exhibited excellent linearity over the concentration range of 0.00412–0.02472 mg mL^−1^ with a correlation coefficient of R^2^ = 0.9998, demonstrating its suitability for quantitative analysis.

Among the numerous bioactive compounds present in propolis, flavonoids such as galangin have received considerable attention due to their antioxidant, cytoprotective, and pharmacological properties. Galangin has been reported to exhibit antioxidant and antigenotoxic effects in mammalian cells, demonstrating its ability to modulate oxidative stress-related mechanisms while maintaining cellular protection under appropriate conditions [69,70,71]. Recent studies have further highlighted the therapeutic potential of galangin as a food-derived flavonoid with broad biological activity, including antioxidant, anti-inflammatory, antimicrobial, and protective effects against various pathological processes [72,73,74,75,76,77]. The biological activity of galangin and other propolis-derived polyphenols has contributed to increasing interest in their incorporation into advanced biomaterials and functional coatings, where controlled immobilization may provide sustained biological functionality while minimizing potential adverse effects.

The biological effects of propolis are primarily associated with its complex mixture of polyphenols, including flavonoids and phenolic acids, which act through multiple molecular pathways. Recent reviews have described the anticancer, antioxidant, and immunomodulatory mechanisms of propolis constituents, including regulation of oxidative stress pathways, apoptosis, inflammatory signaling, and cellular defense mechanisms [70,71,72,73]. However, the activity of propolis cannot be attributed exclusively to individual compounds, since synergistic interactions between multiple constituents contribute to its overall biological profile. The composition of propolis varies considerably depending on botanical origin and geographical location, resulting in differences in phenolic profiles and biological activity [74,76]. This variability highlights the importance of chemical characterization when developing propolis-containing functional materials, as differences in extract composition may directly influence material performance and biological response.

Polyphenolic compounds exhibit complex redox behavior and may function as both antioxidants and pro-oxidants depending on their concentration, chemical structure, and cellular environment. Although flavonoids are commonly associated with antioxidant activity due to their ability to scavenge reactive oxygen species and donate hydrogen atoms, under certain conditions they may generate reactive intermediates or influence cellular redox signaling pathways [78,79,80]. Therefore, interpretation of antioxidant activity and biological effects requires consideration of concentration-dependent responses and the specific experimental conditions. The structural characteristics of propolis-derived polyphenols, including hydroxyl group arrangement and electron delocalization, strongly influence their radical-scavenging capacity and biological activity [22].

In the present study, the incorporation of propolis extracts into chitosan- and alginate-based textile coatings was accompanied by cytotoxicity evaluation to assess their suitability for potential skin-contact applications. The observed cell viability results indicate that the prepared coatings maintained acceptable biocompatibility under the applied experimental conditions, supporting the potential of propolis-based biopolymer systems as functional textile materials. However, considering the complex biological activity of propolis constituents and the concentration-dependent effects reported in the literature, further investigations involving different cell models, longer exposure periods, and expanded biological assays are necessary to fully characterize the safety profile of these materials. The combination of chemical characterization, morphological evaluation, and biological assessment provides a comprehensive approach for the rational development of sustainable propolis-based coatings with potential applications in healthcare textiles and topical biomedical products.

Therefore, the results indicate that the investigated coated samples maintained acceptable cell viability under the applied test conditions, suggesting their potential suitability for skin-contact applications. It should be noted that the present cytotoxicity evaluation represents an initial biocompatibility assessment. Additional studies involving increased numbers of biological replicates, statistical evaluation, different exposure periods, and complementary biological assays will be required for a comprehensive evaluation of the safety and performance of the developed functional textiles.

Overall, the combination of HPLC, FTIR, SEM, and cytotoxicity analyses provides complementary evidence supporting the proposed propolis–biopolymer coating strategy. HPLC analysis established clear compositional differences between the hydroalcoholic and olive oil extracts, particularly regarding the concentration of bioactive flavonoids such as galangin, thereby providing a chemical basis for interpreting differences in coating behavior. FTIR spectroscopy confirmed the successful incorporation of the propolis-containing biopolymer coatings onto the textile substrate through the identification of characteristic functional groups and interactions between the coating components and the textile surface. SEM observations further verified the presence of the deposited coatings and revealed qualitative differences in surface morphology that were consistent with both the chemical composition of the extracts and the physicochemical properties of the extraction media. Finally, cytotoxicity testing demonstrated that all coated samples maintained cell viability above the threshold specified in ISO 10993-5, indicating that the developed formulations are cytocompatible under the applied experimental conditions. Taken together, these complementary analytical techniques provide a coherent and mutually supportive evaluation of the developed functional coatings, demonstrating that the selected extraction procedures, biopolymer matrices, and coating formulations successfully produced bioactive textile surfaces with favorable morphological, chemical, and biological characteristics. Although additional studies, including quantitative surface characterization, antimicrobial evaluation, and long-term biological performance testing, are required before practical implementation, the present results establish a robust scientific foundation for the further development of propolis-based functional textiles intended for skin-contact and wound-care applications.

## 3. Materials and Methods

### 3.1. Materials

Raw propolis was collected from private apiaries located in the Gračani area on the slopes of Medvednica Mountain near Zagreb, Croatia, and from the Sveta Jana region, Croatia. 

Ethanol (food-grade ethanol), distilled water, extra virgin olive oil, and lavender essential oil distilled from natural plants collected in Croatia were used as extraction media. Textile substrates and biopolymers employed for coating preparation are described in Section 2.3.

A reference cotton textile substrate (100% cotton) was used in this study. The fabric was supplied by SDC Enterprises Ltd. (Bedford, UK) and is designed for textile testing according to HRN ISO 105-F standards.

**Figure 6:**
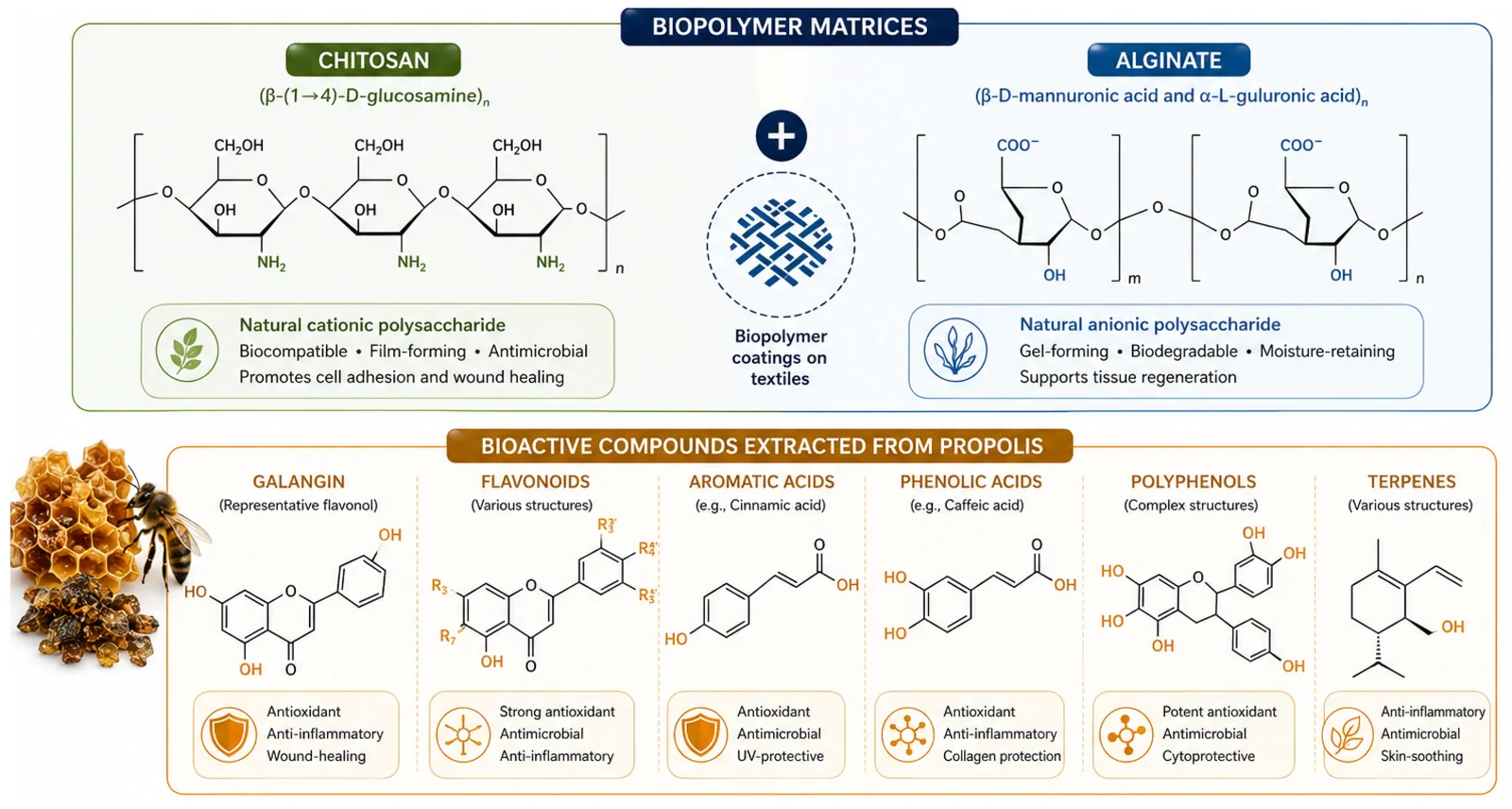
Biopolymer matrices (chitosan and alginate) and bioactive compounds (galangin, flavonoids, aromatic acids, phenolic acids, polyphenols, terpenes) investigated in this work.

Chitosan (deacetylated, Mw ≈ 1526.45 g/mol, CAS: 9012-76-4), glycerol (CAS: 56-81-5), and acetic acid (CAS: 64-19-7) were purchased from Sigma Aldrich, Burlington, MA, USA, and used for preparation of polymeric coating systems. Sodium alginate (low viscosity, CAS: 9005-38-3) was purchased from Thermo Scientific, Waltham, MA, USA. Biopolymer matrices and bioactive compounds extracted from propolis, used in this work are presented in Figure 6.

### 3.2. Preparation of Propolis Extracts

Propolis extraction was performed between 2 December 2025, and 8 January 2026. The extraction procedure was based on the Croatian Regulation on the Quality of Honey and Other Bee Products (Official Gazette NN 20/2000, Article 15), which specifies that propolis placed on the market must contain at least 35% alcohol-extractable substances. 

Propolis dissolution was performed according to Article 15 of the Ordinance on the Quality of Honey and Other Bee Products (Official Gazette 20/2000; https://narodne-novine.nn.hr/clanci/sluzbeni/2000_02_20_280.html, assessed in 20 May 2026), which specifies that propolis placed on the market must contain at least 35% of alcohol-extractable substances. Based on preliminary results and maintaining proportional scaling of the formulation (350 g propolis per 1000 mL total volume), two working solutions were prepared: Solution I consisted of 87.5 g propolis, 70 mL water, and 180 mL food-grade ethanol (C_2_H_5_OH), while Solution II consisted of 43.75 g propolis, 125 mL olive oil, and 8 drops of lavender essential oil.

Solution I was designed as a hydroalcoholic system to ensure efficient extraction of a broad range of propolis bioactive compounds. Ethanol is known to extract significantly higher amounts of flavonoids and total phenolic compounds compared to water [21], and propolis ethanol extracts are typically enriched in resinous components, flavonoids, phenolic acids, and their esters, making ethanolic extracts the standard form used in pharmacological studies [22]. Although water also contributes to the extraction of polar phenolic compounds, their concentrations are generally lower than in ethanol-based systems [23], while waxes are largely insoluble in water and most lipophilic constituents are preferentially soluble in ethanol [22]. The combined ethanol–water system was therefore used to enhance extraction efficiency across a wider polarity range of bioactive constituents [24].

Solution II represented an alternative, oil-based extraction approach aimed at preserving the full spectrum of propolis bioactive compounds. Olive oil was used as a non-polar extraction medium capable of solubilizing selected flavonoids and phenolic acids; however, reported extraction efficiencies for total phenolics are generally lower than those achieved with ethanol, particularly under room-temperature conditions. Nevertheless, previous studies have shown that oil-based propolis extracts may contain flavonoids such as naringenin, galangin, and kaempferol, as well as phenolic acids. Some authors further suggest that the lower polarity of oil-based systems can facilitate the extraction of a broader range of compounds, despite the overall lower total phenolic yield compared to ethanolic extracts [25].

**Figure 7:**
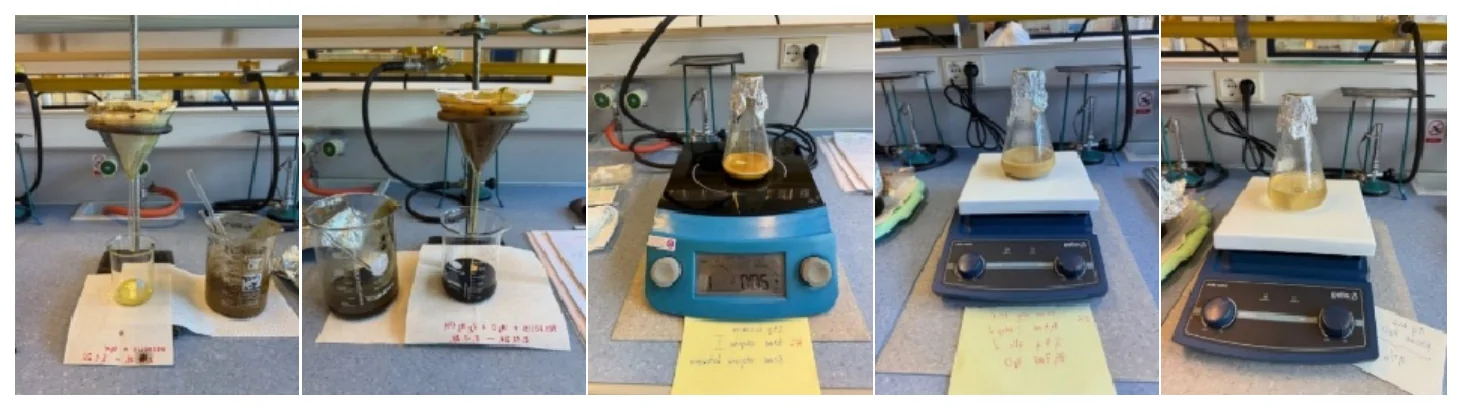
Preparation of propolis extracts.

Different extraction systems were prepared to obtain extracts with different chemical compositions and to evaluate their suitability for textile coating formulations (Figure 7).

#### 3.2.1. Hydroalcoholic Propolis Extract (Extract I)

The first extraction system consisted of 87.5 g of raw propolis, 70 mL of distilled water, and 180 mL of food-grade ethanol, corresponding to a scaled-down formulation derived from the proportions specified in the Croatian regulation.

A mixture of ethanol and water was selected as the extraction medium to facilitate the extraction of a broad spectrum of bioactive compounds present in propolis. Ethanol is known to efficiently dissolve flavonoids, phenolic acids, their esters, and resinous constituents, while the presence of water promotes the extraction of more polar compounds and contributes to an increased overall extraction yield. The hydro-alcoholic system was therefore expected to provide a balanced extract containing both lipophilic and hydrophilic bioactive components.

The extraction mixture was stored in a closed container at room temperature and protected from direct light throughout the extraction period. After extraction, the resulting solution was separated from insoluble residues and used for further analyses and coating preparation.

#### 3.2.2. Olive Oil Propolis Extract (Extract II)

The second extraction system was developed as an alternative extraction approach intended to preserve and recover bioactive constituents of propolis using a lipid-based medium. This formulation contained 43.75 g of raw propolis, 125 mL of olive oil, and eight drops of lavender essential oil.

Olive oil was selected as an alternative extraction medium because it can dissolve certain flavonoids and phenolic acids while avoiding the use of alcohol. Previous studies have shown that olive oil extracts generally contain lower concentrations of total phenolic compounds than ethanolic extracts; however, they can retain specific bioactive compounds, including flavonoids such as naringenin, galangin, and kaempferol, as well as selected phenolic acids. The lower polarity of the oil-based system may facilitate the extraction of certain lipophilic constituents that are less efficiently recovered by aqueous extraction systems.

The extraction mixture was maintained under the same storage conditions as Extract I until further use.

### 3.3. Preparation of Propolis–Biopolymer Textile Coatings

Textile coatings were prepared by incorporating the prepared propolis extracts into biopolymer solutions. The coating formulations were applied to textile substrates using a deposition technique designed to achieve uniform coverage of the fabric surface. Following application, the coated samples were dried under controlled laboratory conditions, to efficiently functionalize materials with proven antimicrobial compounds and optimized conditions, based on our previous investigations [26,27,28,29].

Attention was paid to coating formation and solvent evaporation processes because these parameters influence the final morphology, homogeneity, and distribution of active compounds within the coating layer. The occurrence of the coffee-ring effect during drying was monitored and evaluated as a factor affecting coating uniformity.

GLYMO (3-glycidyloxypropyltrimethoxysilane, 98%; Sigma-Aldrich, St. Louis, MO, USA) was hydrolyzed either with distilled water alone or with hydrochloric acid (37%; Sigma-Aldrich) as an acidic catalyst. Hydrochloric acid was selected because of its volatility, allowing its removal by evaporation before or during the condensation process. Based on previous studies [17], two GLYMO-based formulations were selected for further investigation. Treatment I consisted of GLYMO hydrolyzed with distilled water at a GLYMO-to-water ratio of 1:3, while Treatment II employed hydrochloric acid-catalyzed hydrolysis with a GLYMO-to-water ratio of 1:1.5. c. GLYMO was added dropwise, and the mixture was stirred for 1 h at 20 °C. The resulting sol was transferred into Teflon vessels for dip-coating. 

Cotton fabric samples (5 × 5 cm) were coated using a dip-coating process at withdrawal speeds of 0.5, 1.0, 1.5, and 2.0 mm s^−1^. Dip-coating, also known as chemical bath deposition (CBD), was selected as a simple and cost-effective technique for depositing thin nanostructured coatings.

Following deposition, the coated samples were allowed to gel at room temperature for 24 h and were subsequently dried at 100 °C for 1 h. No additional catalysts were used to promote epoxy-group polymerization. Partial polymerization was intentionally maintained to limit excessive crosslinking and preserve the flexibility and softness of the organic phase. 

### 3.4. Fourier Transform Infrared Spectroscopy (FTIR)

Fourier transform infrared spectroscopy (SHIMADZU, IRTracer-100, Tokyo, Japan) was used to investigate the chemical composition of the coatings and to identify interactions between propolis constituents, biopolymers, and textile fibers. Spectra were recorded over the appropriate infrared range, and characteristic absorption bands corresponding to hydroxyl, carbonyl, aromatic, and other functional groups were analyzed.

### 3.5. Scanning Electron Microscopy (SEM)

The surface morphology of the coated textile samples was examined using scanning electron microscopy (SEM) using a Tescan Vega III Easyprobe (Brno, Czech Republic). Micrographs were obtained at various magnifications to evaluate coating coverage, surface uniformity, coating thickness, and the distribution of deposited material on textile fibers. Particular attention was given to morphological features associated with drying phenomena and coffee-ring formation.

### 3.6. High-Performance Liquid Chromatography (HPLC) 

The chemical composition of the prepared propolis extracts was analyzed using high-performance liquid chromatography (HPLC, LC-2030C NT, SHIMADZU, Tokyo, Japan). The method was employed to identify and quantify characteristic phenolic compounds and flavonoids present in the extracts. Chromatographic profiles of hydroalcoholic and oil-based extracts were compared to assess differences in extraction efficiency and compound selectivity.

Galangin was selected as a representative marker for evaluating the propolis extracts because it is one of the characteristic flavonoids widely detected in propolis samples from different geographical regions [31]. Its frequent occurrence, together with its well-established chemical structure and biological relevance, makes it a suitable indicator for comparing the efficiency of different extraction approaches. Although propolis contains a complex mixture of phenolic compounds and flavonoids, the determination of galangin content provides valuable information about the relative extraction capacity of the applied solvent systems. In this study, differences in galangin concentration between hydroalcoholic and oil-based extracts reflected variations in the ability of these extraction media to recover bioactive flavonoid constituents, thereby contributing to the observed differences in coating composition and morphology.

The analytical procedure was successfully transferred to and validated in the laboratory of the Croatian Institute of Public Health (HZJZ), where it was applied for the quantitative determination of galangin in the investigated propolis samples.

Subsequently, the galangin content of the prepared propolis extracts was determined in order to evaluate the extraction efficiency of the different extraction media and to compare the obtained extracts with a commercially available propolis preparation.

The following samples were submitted for HPLC analysis: extract I (hydroalcoholic propolis extract), extract II (olive oil-based propolis extract), a commercially available propolis extract used as a reference sample. Based on the analytical results, the sample designations were: sample **1-1**—Extract I (hydroalcoholic extract), sample **2-1**—Extract II (olive oil extract), sample **A-1**—commercially available propolis extract.

Propolis represents a highly complex natural material whose chemical composition and biological activity are strongly influenced by botanical origin, geographical location, and extraction methodology. Recent advances in extraction technologies have demonstrated that the recovery of bioactive constituents from propolis depends significantly on solvent polarity, extraction conditions, and the physicochemical characteristics of individual compounds [48]. Although hundreds of compounds have been identified in propolis, including flavonoids, phenolic acids, terpenoids, and aromatic derivatives, polyphenolic compounds are considered the main contributors to its biological effects [49]. Studies on Mediterranean and Croatian propolis samples have demonstrated considerable chemical diversity, with variations in phenolic profiles identified by advanced chromatographic and spectroscopic approaches, including GC-MS, FTIR-ATR, and UHPLC-based techniques [52,53].

### 3.7. UV–Visible Spectroscopy

UV–Visible spectroscopy Lambda 20, Perkin Elmer Corporation, Massachusetts, USA, was used to measure samples.

### 3.8. Cytotoxicity Assessment

The biocompatibility of the coated textile samples was evaluated by in vitro cytotoxicity testing. 

Human keratinocytes (HaCat, were generous gift from colleague from the University Zagreb) were cultured in Dulbecco’s Modified Eagle’s Medium (DMEM) supplemented with 10% (*v*/*v*) foetal bovine serum (FBS), and 1% (*v*/*v*) penicillin/streptomycin (PenStrep solution). The mentioned culture reagents were obtained from Sigma-Aldrich, Steinheim, Germany. Cell cultures were grown at 37 °C in a 5% CO_2_ atmosphere. The growth medium was replaced every two days. The passage of cells was performed in line with the manufacturer’s recommendations. Cells were detached with 0.25% trypsin/EDTA solution (Sigma-Aldrich, Steinheim, Germany), resuspended, and seeded 24 h prior to experiments.

The MTS test (CellTiter 96^®^ AQueous One Solution Cell Proliferation Assay, Promega, Madison, WI, USA) followed the procedure presented in our previous research [30]. Briefly, 20,000 cells per well were seeded in 96-well transparent plates and exposed for 24 h to textile samples (1–4) after 7-day extraction in cell culture medium supplemented with fetal bovine serum (FBS). Each textile sample (1–4), measuring 5 × 5 cm, was divided into four quadrants: upper left (LG), lower left (LD), upper right (DG), and lower right (DD). Each quadrant was independently immersed in cell culture medium for 7 days to evaluate potential differences in the release of propolis and other incorporated components among different regions of the fabric. After treatment, the cells were washed with PBS to remove residual test samples. The MTS reagent was added to each well, and the cells were re-incubated at 37 °C, in a humidified atmosphere with 5% CO_2_ for 1–4 h, depending on the rate of yellow-violet color development. Lastly, the absorbance was recorded at 492 nm using a microtiter plate reader (Infinite M200PRO, Tecan Group Ltd., Männedorf, Switzerland). The results are presented both as pooled data representing all four quadrants of each textile sample and separately for each individual quadrant. Data were taken from at least two independent experiments (each treatment performed in triplicate) and plotted as a percentage of viable cells to untreated control cells. A 20% DMSO solution was used as positive cytotoxic control.

According to ISO 10993-5, materials with cell viability ≥ 70% were considered non-cytotoxic.

The development of propolis-based functional materials requires a careful balance between biological activity and safety, particularly for applications involving direct contact with human skin. Although propolis and its phenolic constituents are widely recognized for their antioxidant, antimicrobial, and protective effects, their biological responses are strongly dependent on chemical composition, concentration, exposure conditions, and the target biological system. Previous in vitro investigations have demonstrated that propolis extracts may influence cellular responses, but comprehensive evaluation is necessary to distinguish beneficial protective effects from potential cytotoxic or genotoxic effects. For example, Montoro et al. investigated the cytogenetic and genotoxic effects of propolis on human lymphocytes and demonstrated the importance of concentration-dependent evaluation when assessing the biological safety of propolis extracts [68]. These findings emphasize the necessity of performing cytocompatibility studies when incorporating propolis into materials intended for biomedical or skin-contact applications.

### 3.9. Evaluation of the Coffee-Ring Effect

Dino-Light digital microscope (Dunwell Tech, Inc., Los Angeles, CA, USA) imaging was applied in evaluation of coffee-ring effects.

### 3.10. Textile Preparation and Coating Formulation

**Figure 8:**
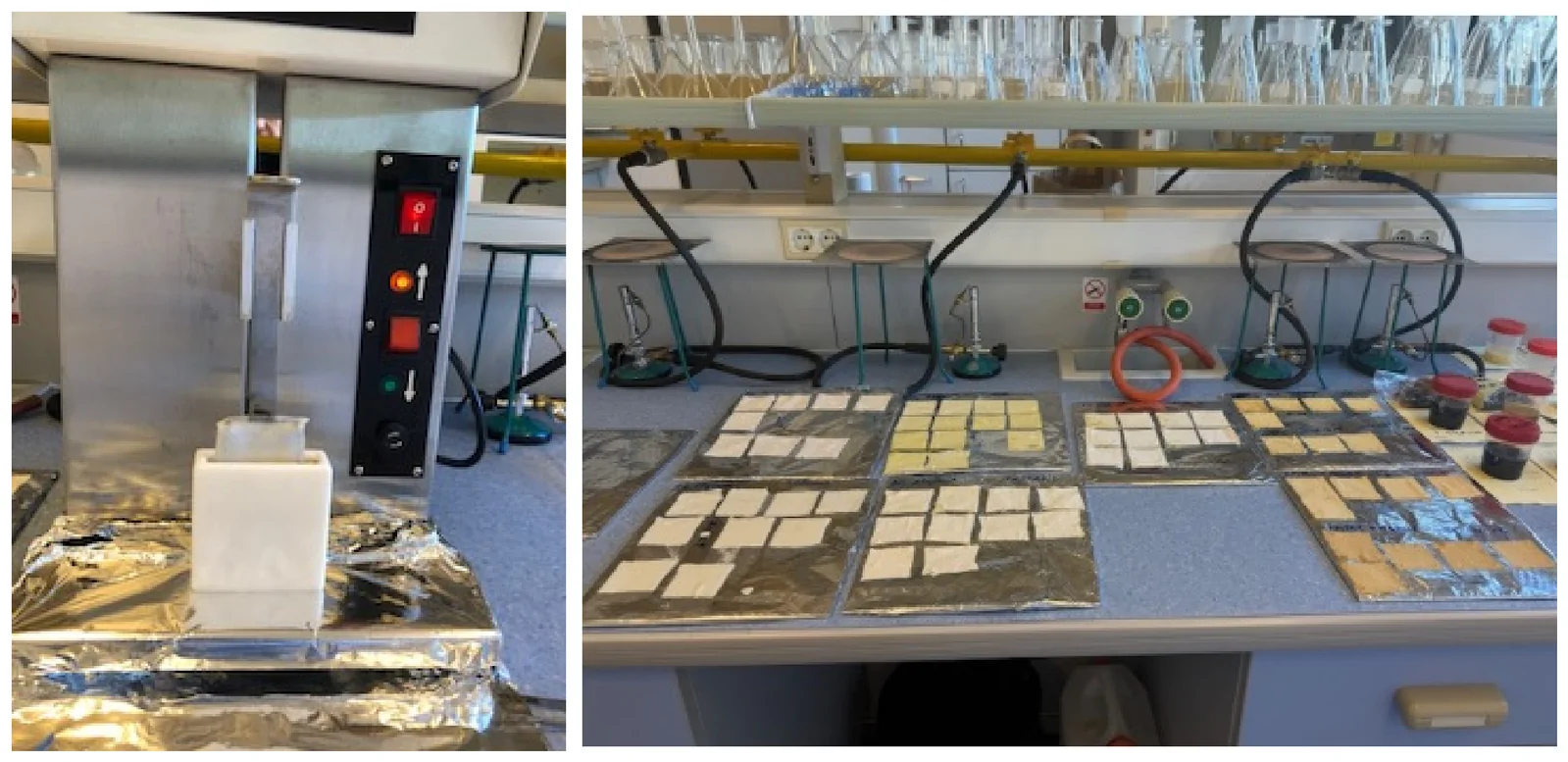
Coating textile samples with different propolis extracts, and drying for 24 h.

Material used in testing was a reference single-component fabric made of 100% cotton fibers, supplied by the authorized manufacturer of textile testing reference materials, SDC Enterprises Limited, UK (fabric intended for testing the color fastness of textile materials to various influences and is produced in accordance with the specifications defined in the latest edition of the HRN ISO 105-F standard series). Samples were cut (5 × 5 cm) and prepared for coating experiments (Figure 8). 

Coating formulations were based on chitosan and sodium alginate matrices. Chitosan solutions (1% *w*/*v*) were prepared by dissolving chitosan in 0.5% (*v*/*v*) acetic acid solution, while sodium alginate solutions (1% *w*/*v*) were prepared in deionized water under continuous stirring until complete dissolution.

Propolis extracts were incorporated into the polymeric matrices at different loadings (0.5%, 5%, and 35% *v*/*v*, relative to the extract content) to assess the effect of bioactive concentration on coating morphology and biological activity.

Glycerol (0.3 g per formulation) was added as a plasticizer to improve film flexibility.

Textile samples were coated via dip-coating, using a controlled withdrawal speed of 1 mm/s. After coating, samples were dried at room temperature for 24 h and subsequently post-cured in a drying oven at 30 °C for 1 h to ensure stabilization of the coating layer.

Formulations were coded as follows: Ic, Ib, Ic: chitosan-based systems with hydroalcoholic extract; IIa, IIb, IIc: chitosan-based systems with oil extract; 1a, 1b, 1c: alginate-based systems with hydroalcoholic extract; 2a, 2b, 2c: alginate-based systems with oil extract; B and C: commercial propolis-based reference formulations. Untreated cotton fabric was used as the control sample. Cut textile samples (5 × 5 cm) were prepared for coating experiments (Figure 8), and coating formulations were based on chitosan and sodium alginate matrices.

## 4. Conclusions

This study demonstrated the successful preparation and characterization of bioactive textile coatings based on propolis incorporated into natural biopolymer matrices for potential topical and biomedical applications. Two extraction systems, namely a hydroalcoholic and an olive oil-based extraction medium, were investigated for the recovery of biologically active propolis constituents and their incorporation into textile coatings.

HPLC analysis confirmed the presence of galangin in both propolis extracts, while FTIR spectroscopy verified the successful incorporation of propolis-derived compounds into the biopolymer coatings. SEM observations demonstrated homogeneous coating deposition on textile fibers and provided valuable insight into the influence of solvent composition on coating morphology and the coffee-ring phenomenon. UV–Vis spectroscopy revealed enhanced ultraviolet absorption properties of the coated textiles, confirming their potential as multifunctional protective materials.

The cytotoxicity assessment using HaCaT keratinocytes demonstrated that all investigated coatings-maintained cell viability above the 70% threshold specified by ISO 10993-5, confirming that none of the formulations exhibited cytotoxic effects. Differences observed among the samples were therefore associated primarily with changes in cellular metabolic activity rather than cytotoxicity. The results further indicated that the type of biopolymer matrix had a greater influence on cell metabolic activity than the type of propolis extract. Chitosan-based coatings consistently preserved or enhanced HaCaT cell viability irrespective of the extraction system, highlighting the excellent cytocompatibility of chitosan and its suitability for skin-contact and wound-care applications.

The hydroalcoholic extraction system exhibited superior efficiency for the recovery of galangin and other phenolic compounds, whereas the olive oil-based extraction system provided a promising alcohol-free alternative capable of preserving lipophilic bioactive constituents. Overall, the obtained results demonstrate that the combination of propolis with natural biopolymers enables the development of safe, bioactive, and multifunctional textile coatings with considerable potential for wound dressings, protective textiles, cosmetic products, and other advanced biomedical applications.

Future research should focus on optimizing the coating formulation and deposition process, improving the uniformity of active compound distribution, investigating the release kinetics of bioactive constituents, and evaluating the antimicrobial, antioxidant, anti-inflammatory, and wound-healing performance of the developed coatings under conditions relevant to their intended applications.

## Figures and Tables

**Table 1:** Sample composition of sample solution I with chitosan, glycerol and acetic acid (HAc) with their FTIR spectra.

Formulation of Solution I		Sample I + 1% CHITOSAN in HAc
I a (35%)	50 mL 1% chitosan 50 mL Sample I 0.3 g glycerol	** 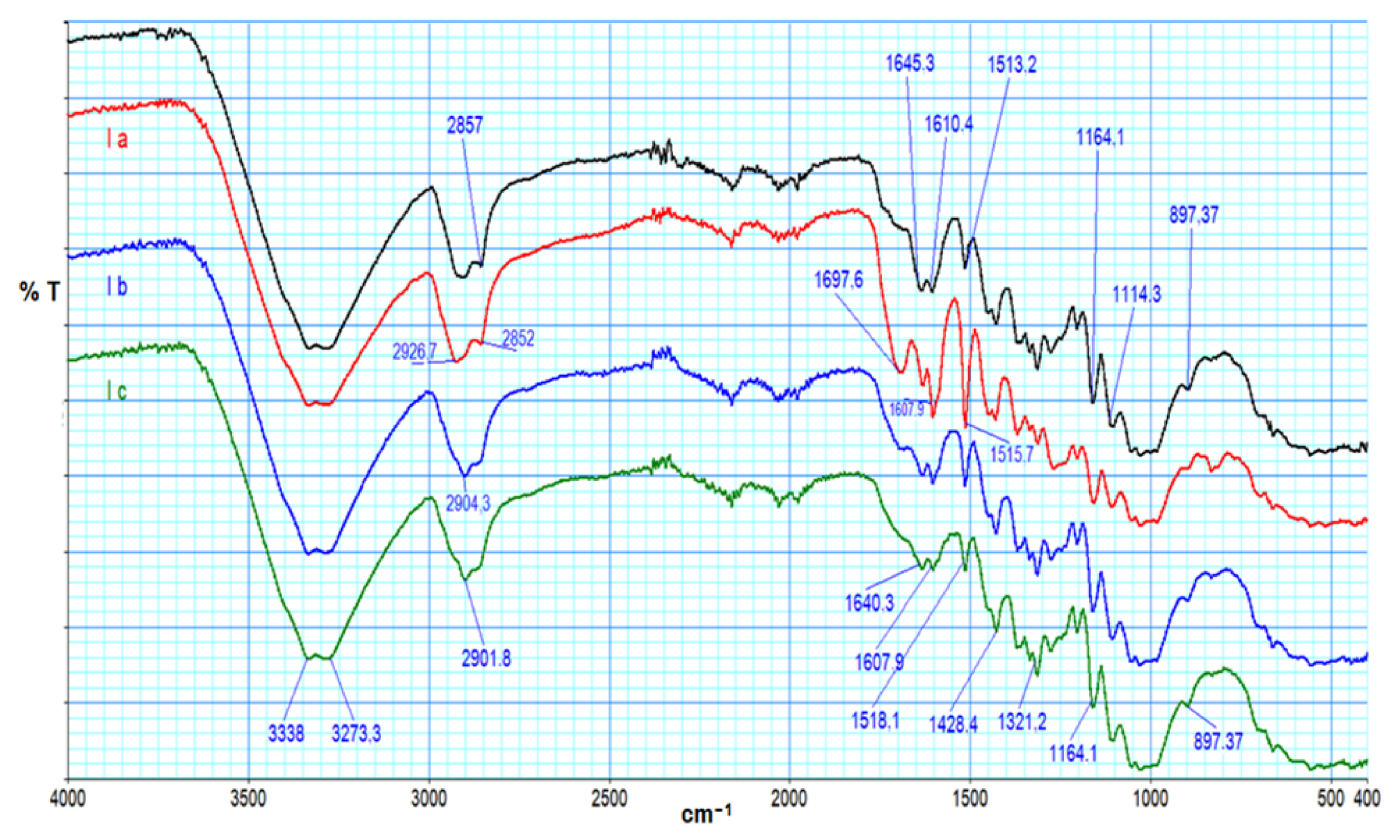 **
I b (5%)	50 mL 1% chitosan 14.3 mL Sample I 0.3 g glycerol 35.7 mL H_2_0	
I c (0.5%)	50 mL 1% chitosan 1.43 mL Sample I 0.3 g glycerol 48.57 mL H_2_0	

**Table 2:** Sample composition of sample solution II with chitosan, glycerol and acetic acid (HAc) with their FTIR spectra.

Formulation of Solution II		Sample II + 1% CHITOSAN (1 g Chitosan–100 mL 1% HAc)
II a (35%)	50 mL 1% chitosan 12.5 mL Sample II 0.3 g glycerol	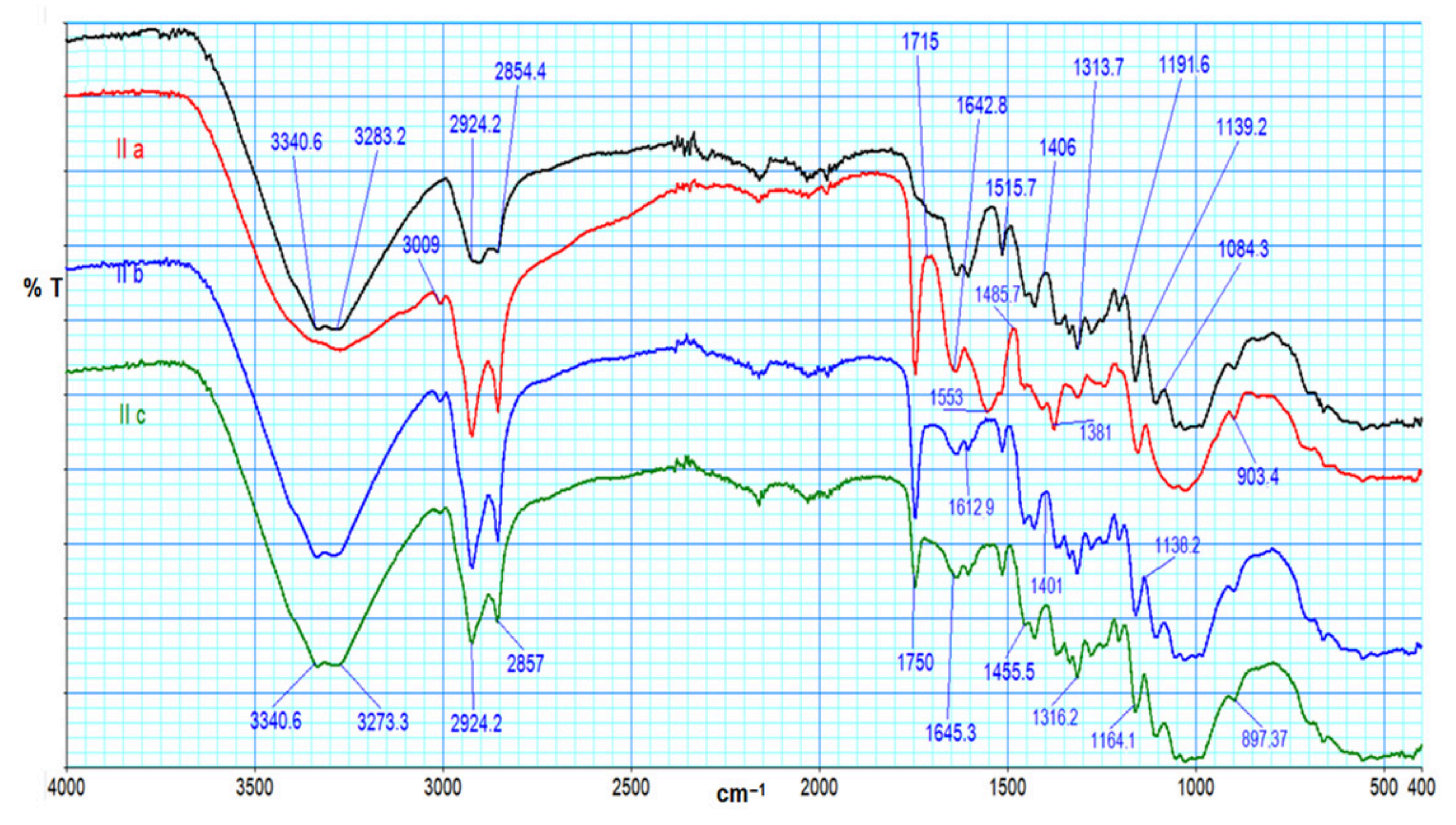
II b (5%)	50 mL 1% chitosan 1.25 mL Sample II 0.3 g glycerol	
II c (0.5%)	50 mL 1% chitosan 0.36 mL Sample II 0.3 g glycerol	

**Table 3:** Sample composition of sample solution I with alginate, glycerol and their FTIR spectra.

Formulation of Solution III		Sample I + 1% ALGINATE (in H_2_O)
1 a (35%)	50 mL 1% alginate 50 mL Sample I 0.3 g glycerol	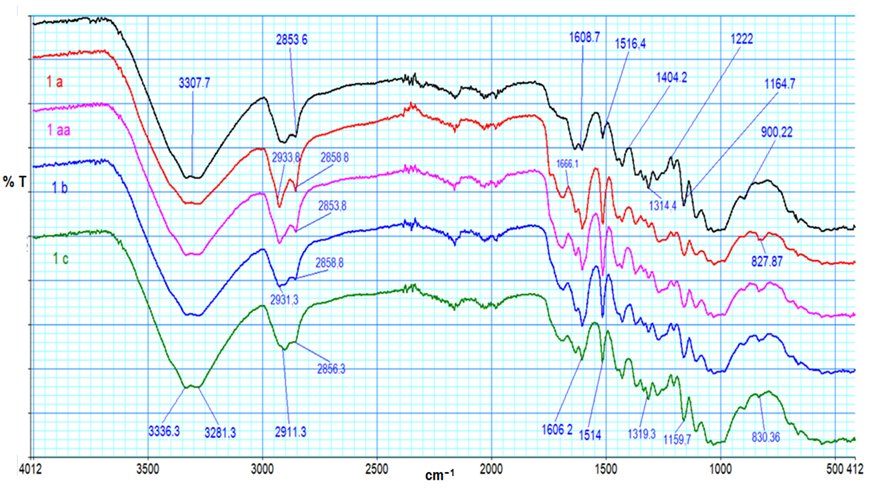
1 aa (35%)	50 mL 1% alginate 50 mL Sample I 0.3 g glycerol 25 mL C_2_H_5_0H,	
1 b (5%)	50 mL 1% alginate 14.3 mL Sample I 0.3 glycerol 35.7 mL H_2_0 35.7 mL C_2_H_5_0H,	
1 c (0.5%)	50 mL 1% alginate 1.43 mL Sample I 0.3 g glycerol 48.57 mL C_2_H_5_0H,	

**Table 4:** Sample solution II with alginate, and glycerol and their FTIR spectra.

Formulation of Solution IV		Sample II + 1% ALGINATE in H_2_O
2 a (35%)	50 mL 1% alginate 12.5 mL Sample II 0.3 g glycerol	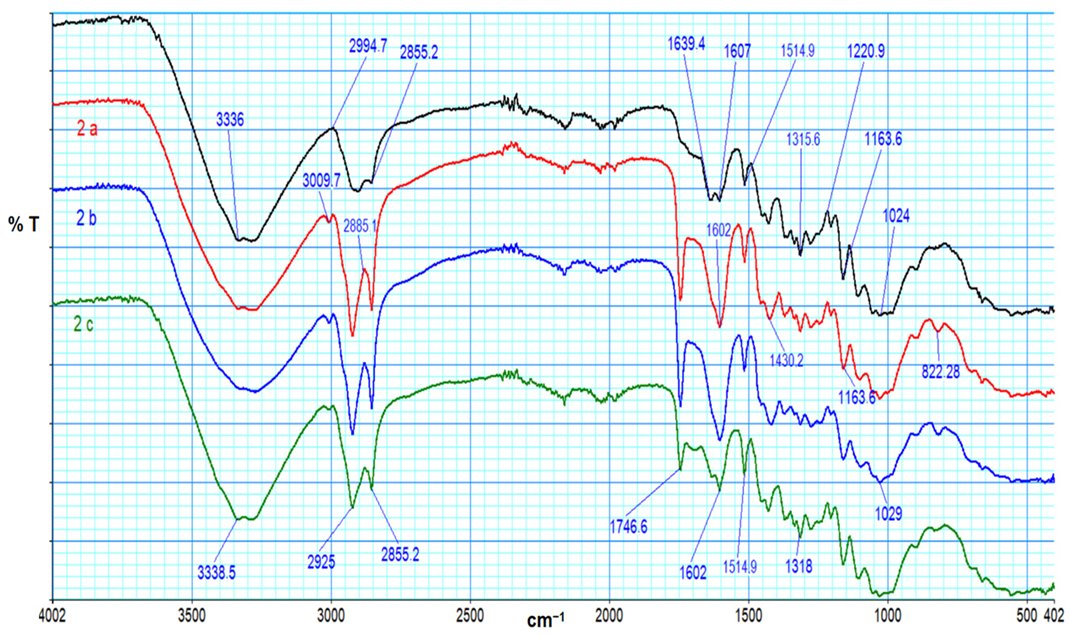
2 b (5%)	50 mL 1% alginate 1.25 mL Sample II 0.3 g glycerol	
2 c (0.5%)	50 mL 1% alginate 0.36 mL Sample II 0.3 g glycerol	

**Table 5:** FTIR spectra and correlated mixtures of two standard propolis extracts (A1 and A2), in mixtures with chitosan (B) and alginate (C).

**A**	**A1 (5%) + A2 (10%)**	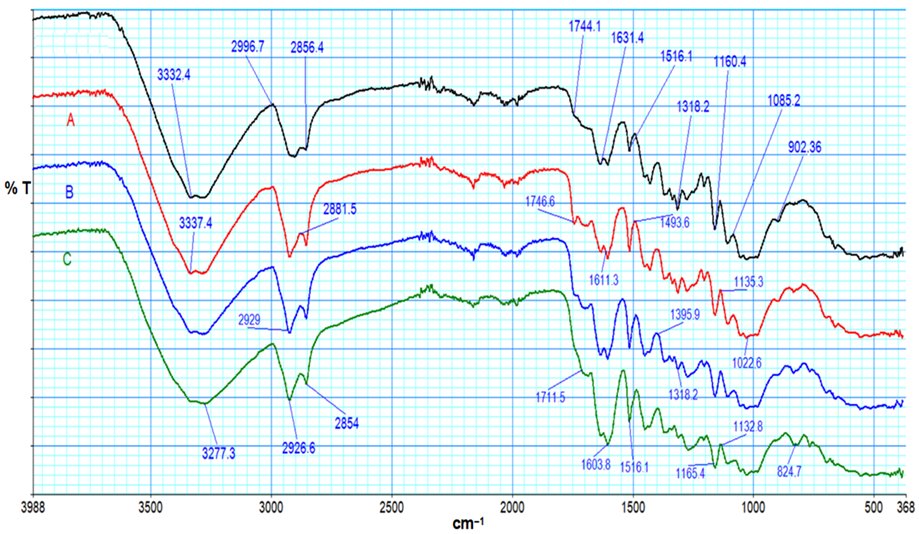
**B**	A1 (5%) + A2 (10%) **0.3 g glycerol** **50 mL 1% chitosan**	
**C**	A1 (5%) + A2 (10%) **0.3 g glycerol** **50 mL 1% alginate**	

**Table 6:** Sample surface SEM microphotographs recorded before modification, investigated under 500 and 2500 magnification.

Sample		500×	2500×
N	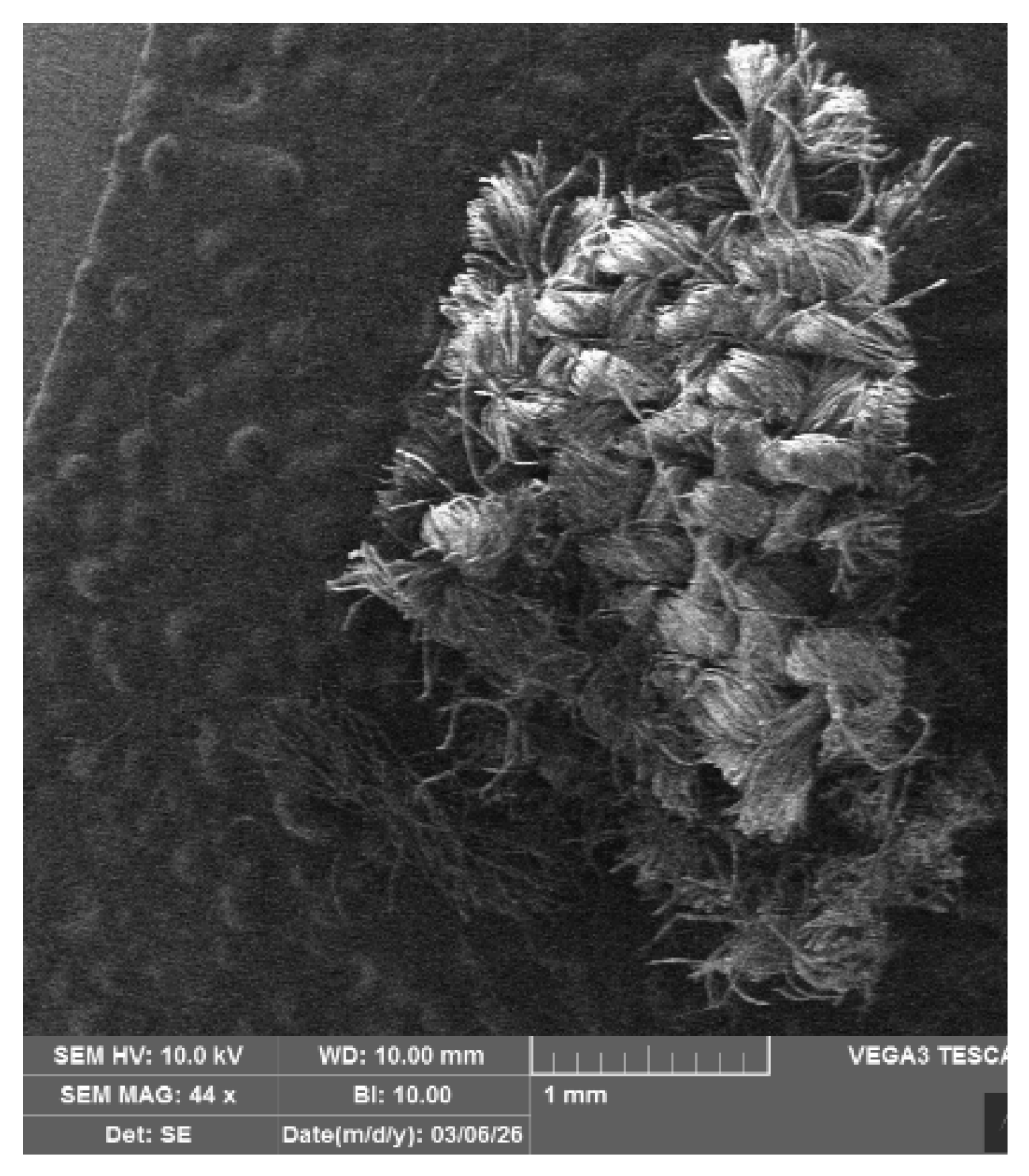	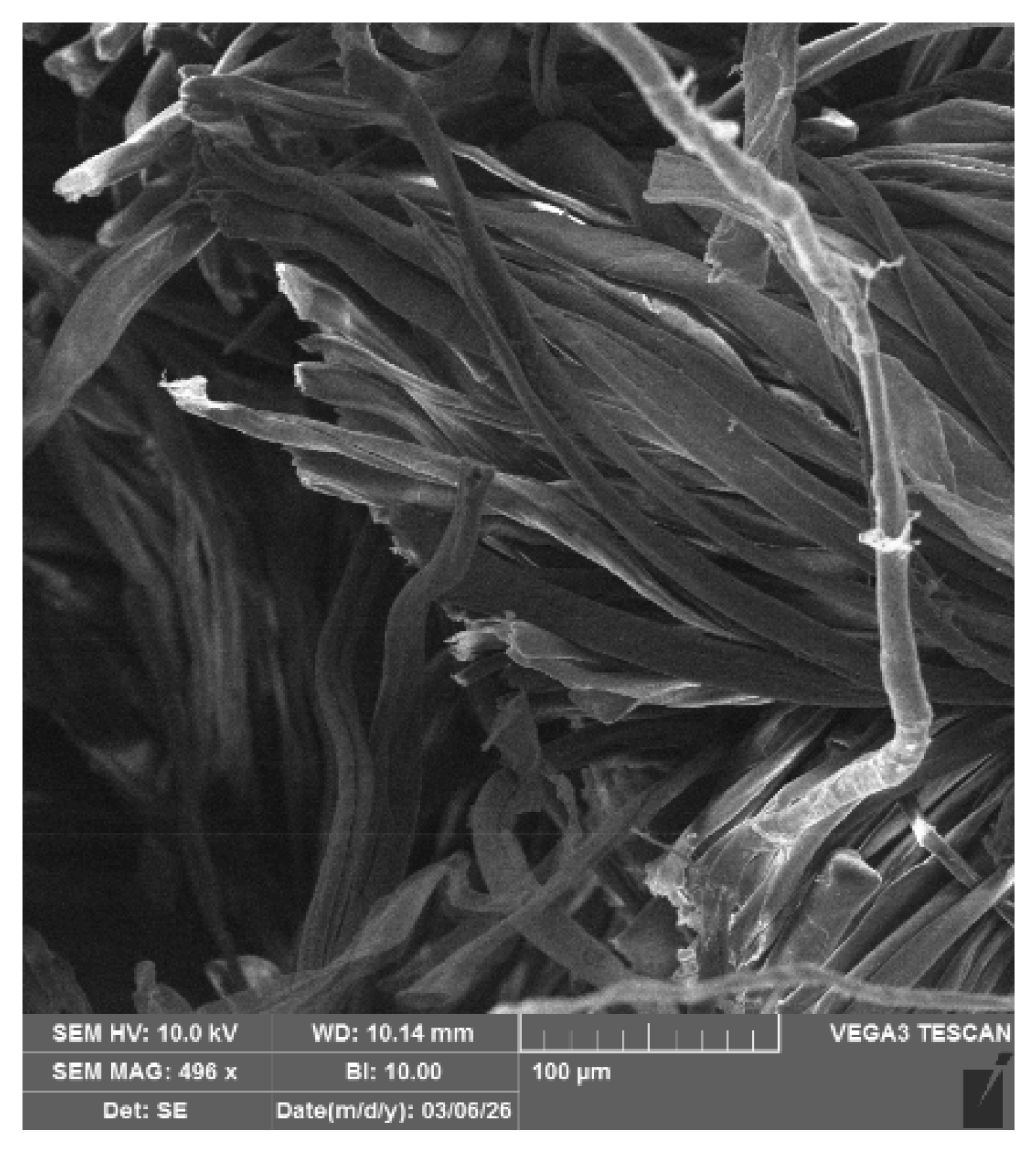	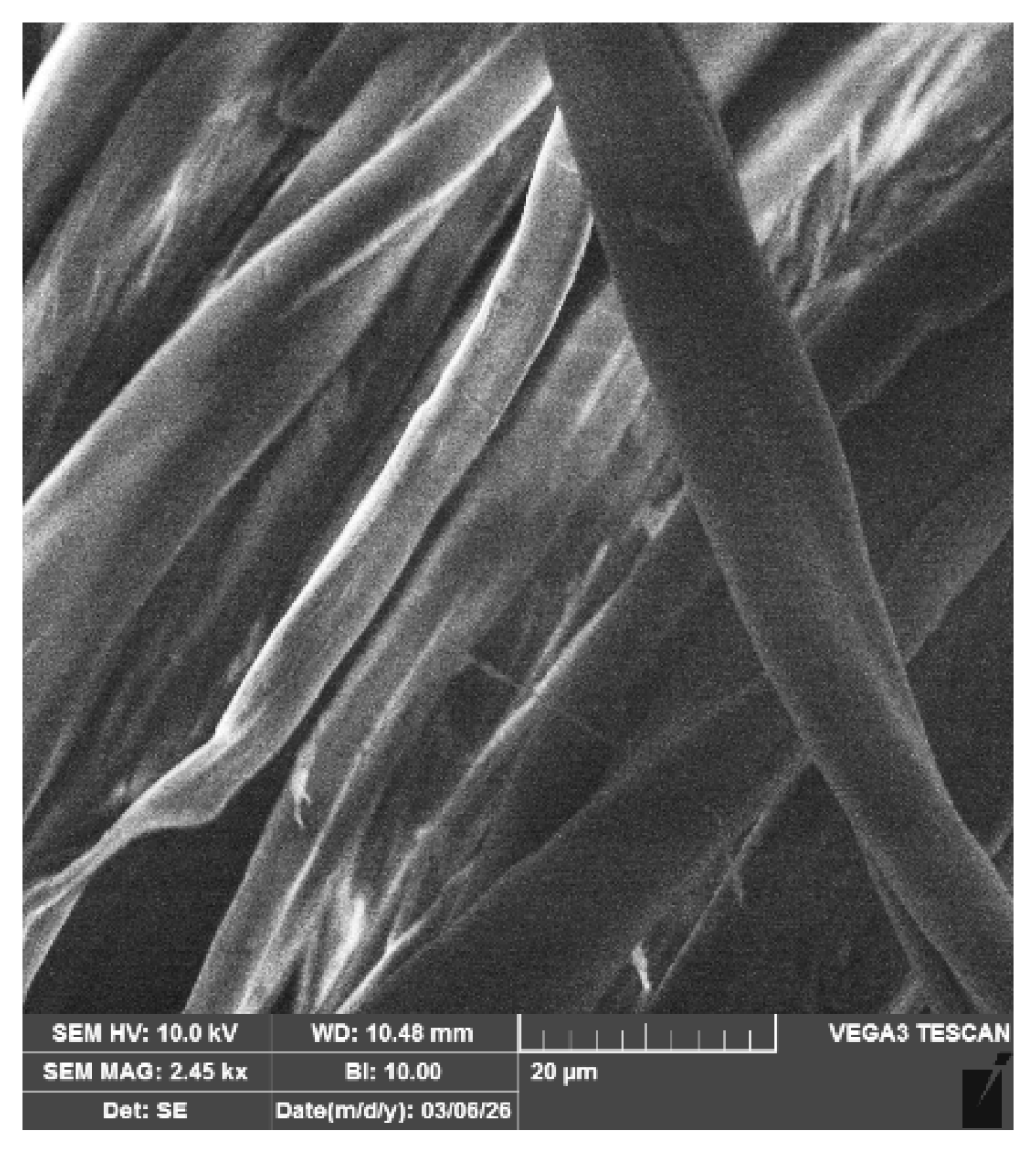
I c	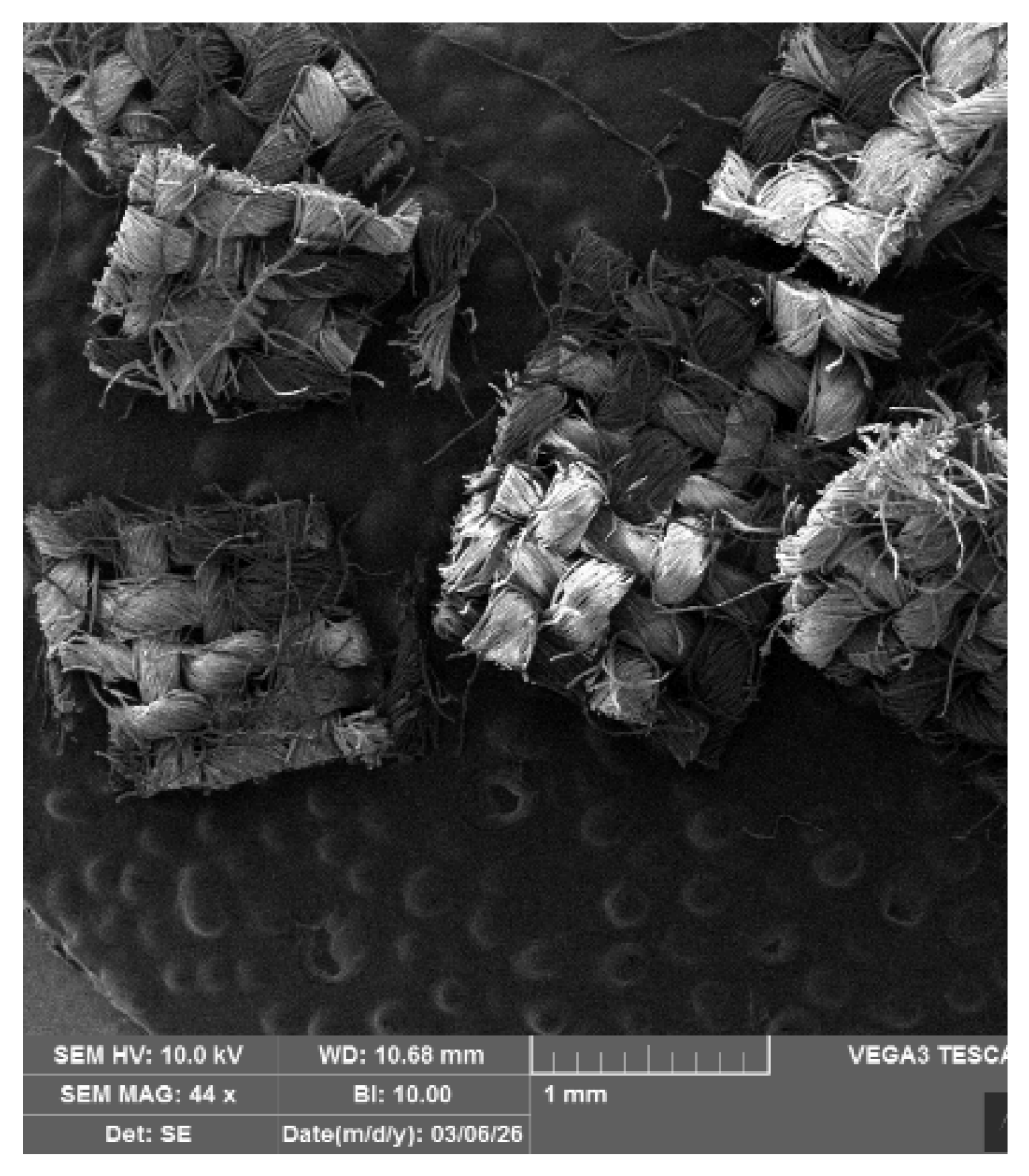	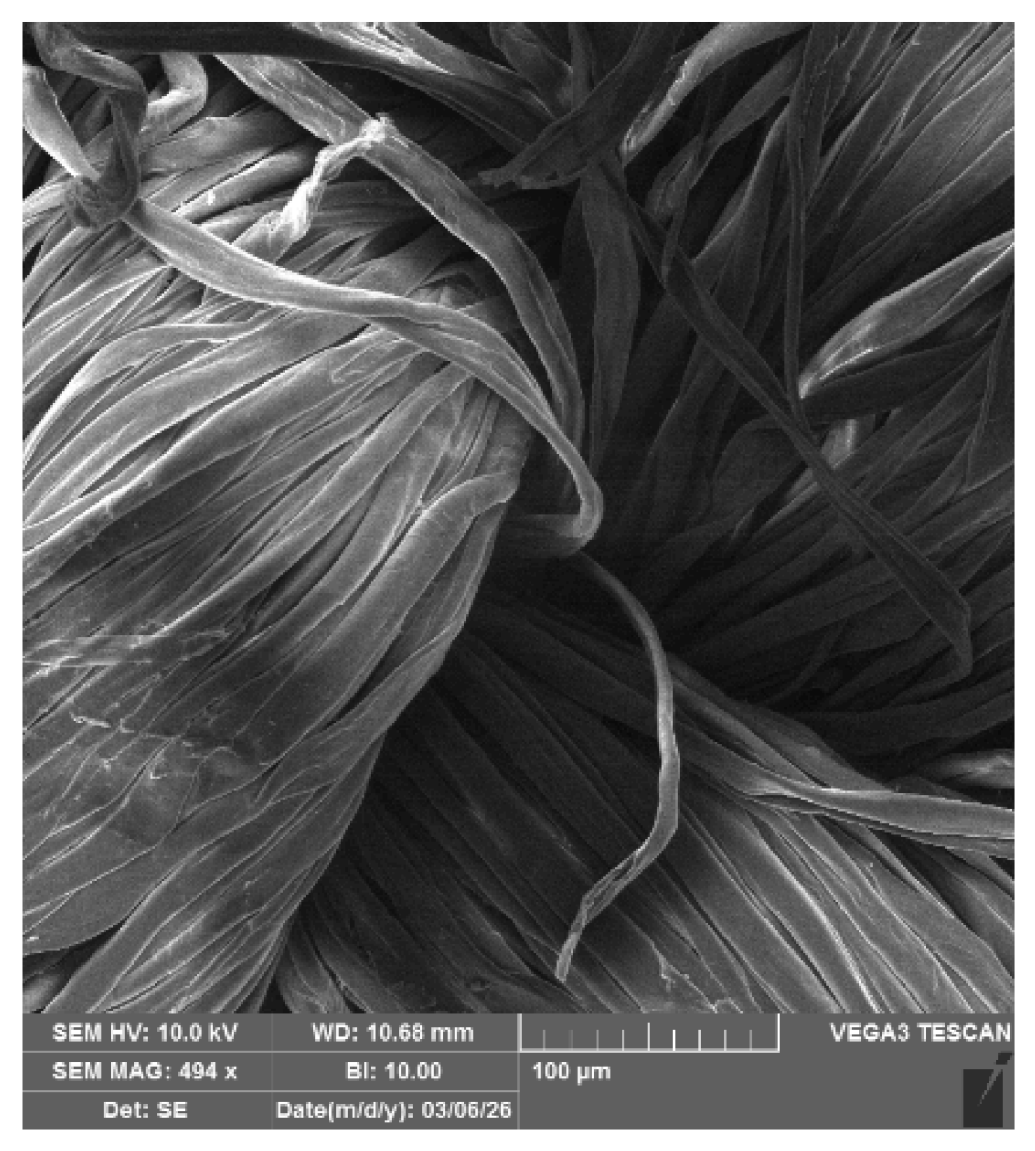	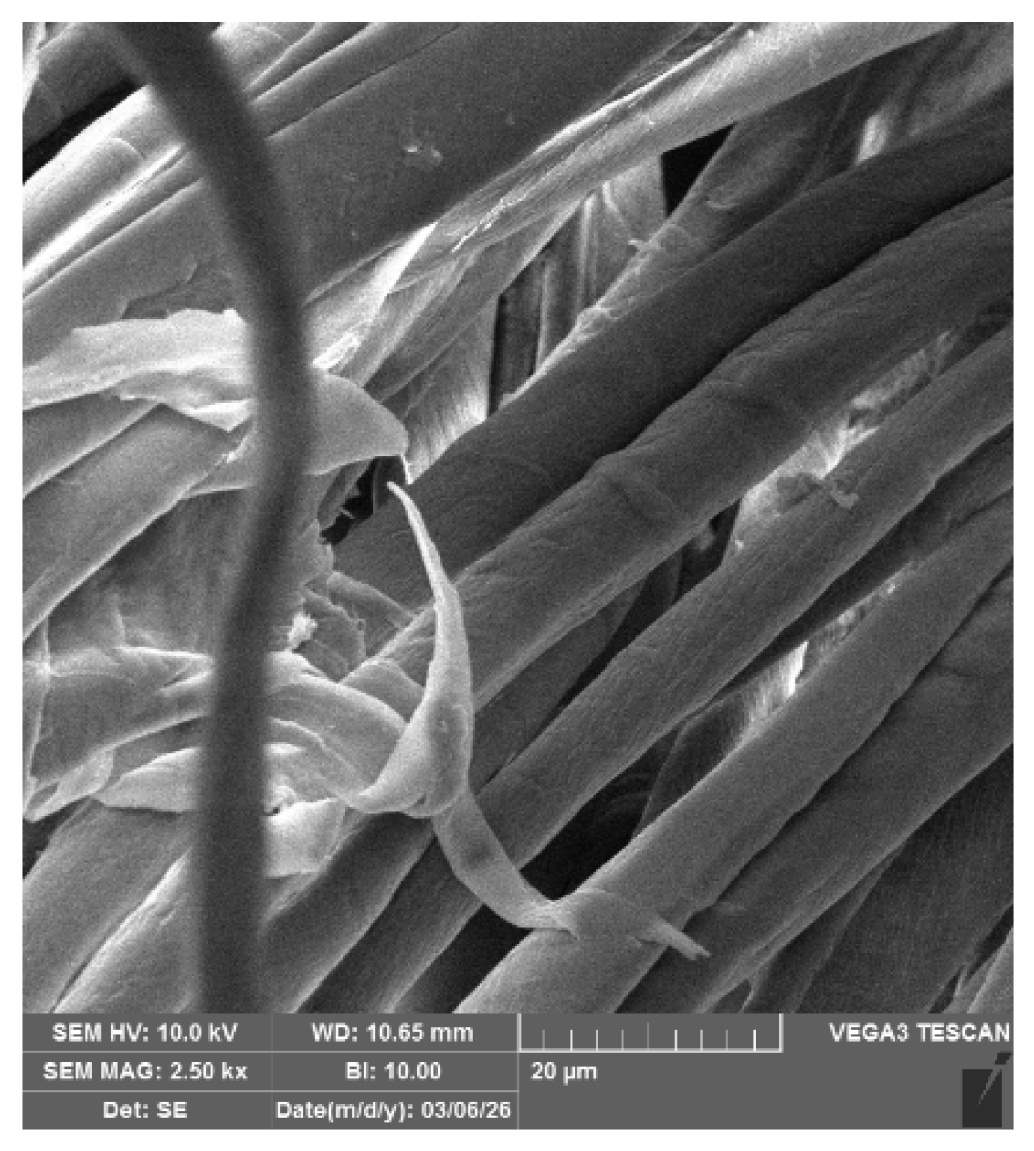
II c	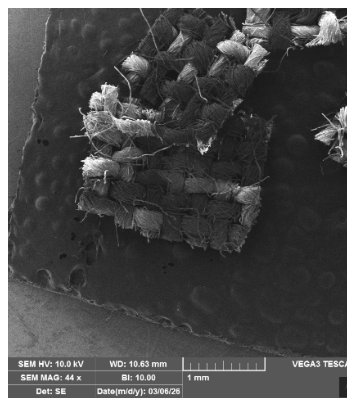	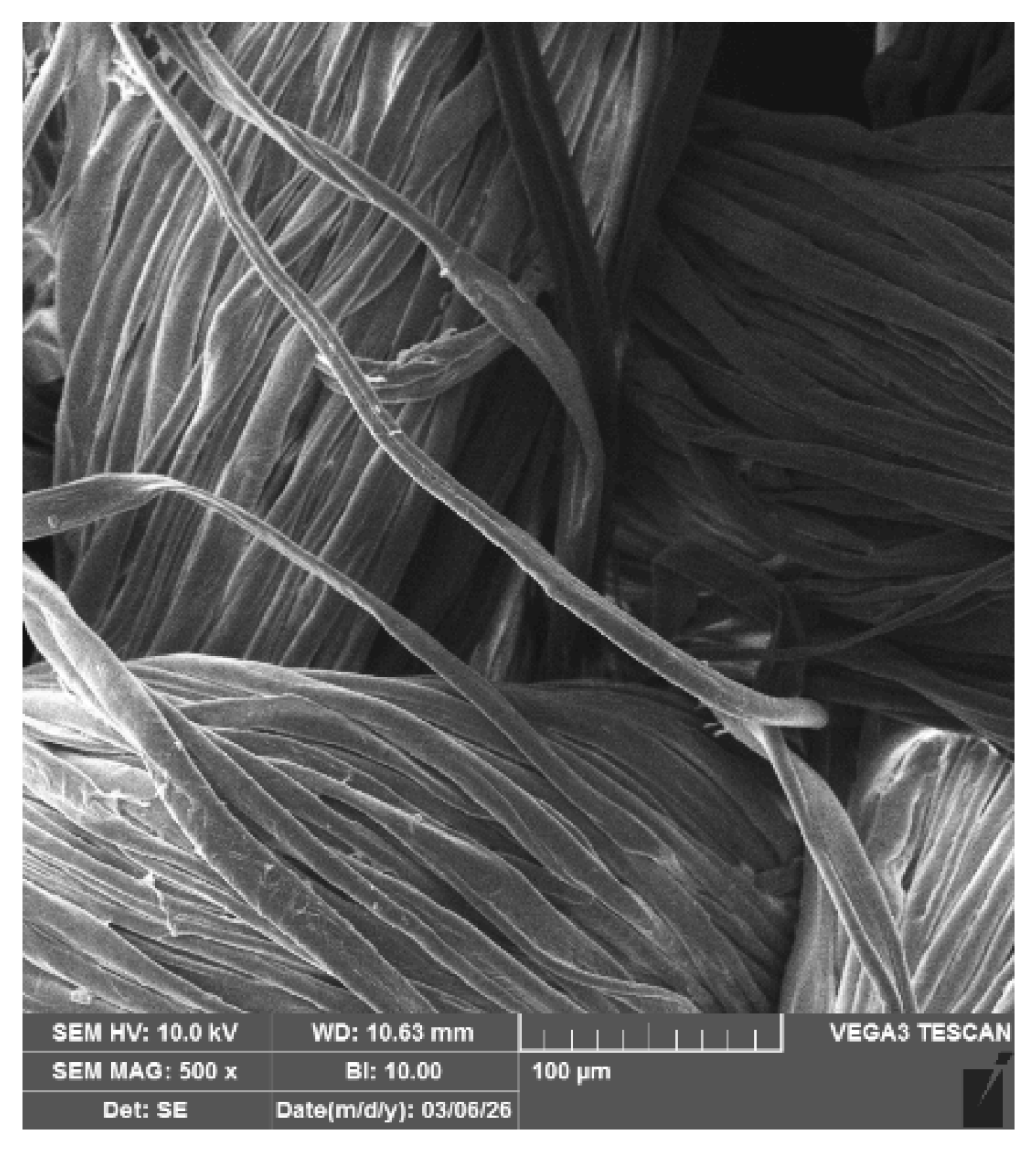	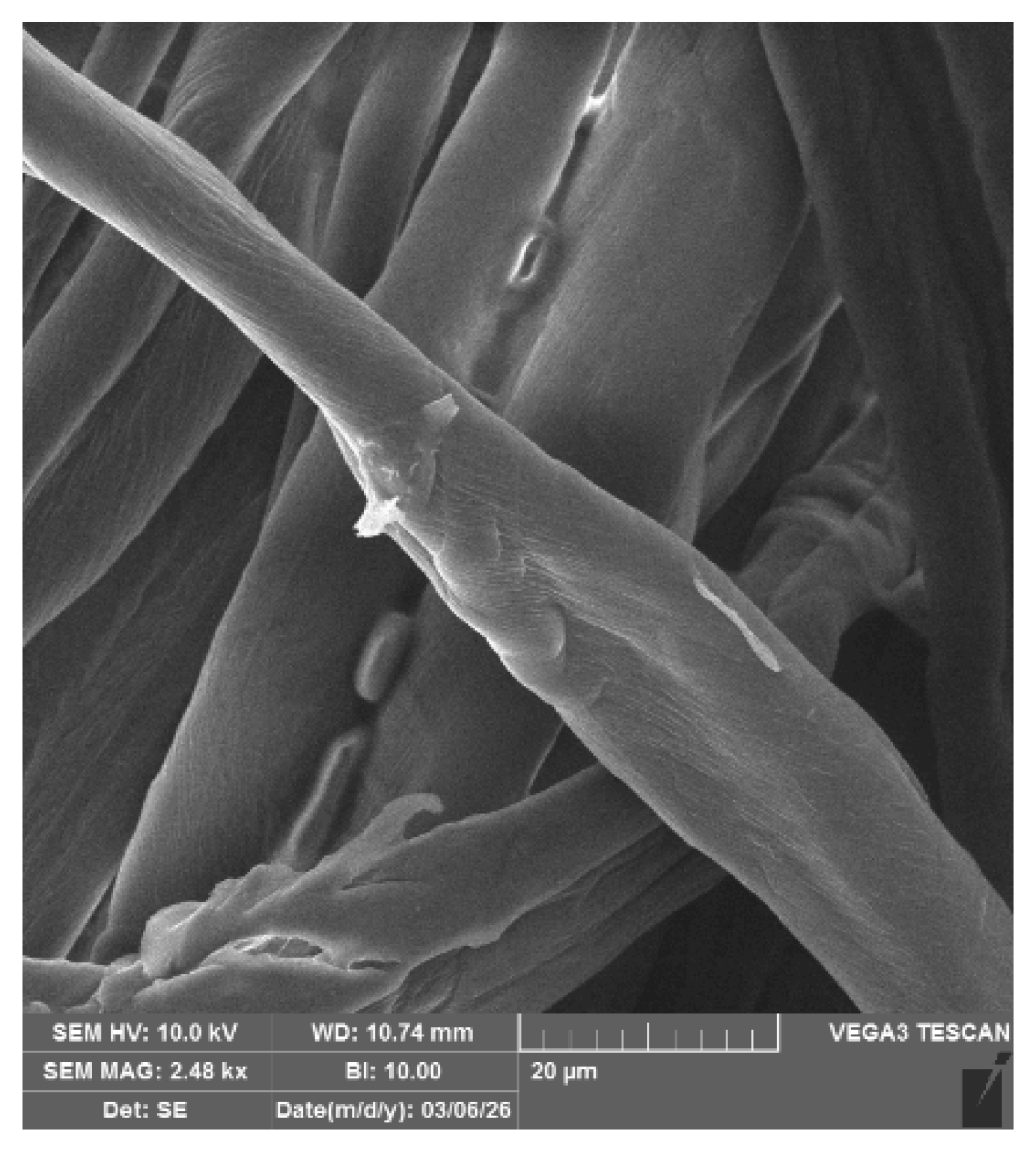

**Table 7:** Sample surface SEM microphotographs recorded after modification, under 500 and 2500 magnification.

Sample		500×	2500×
1 c	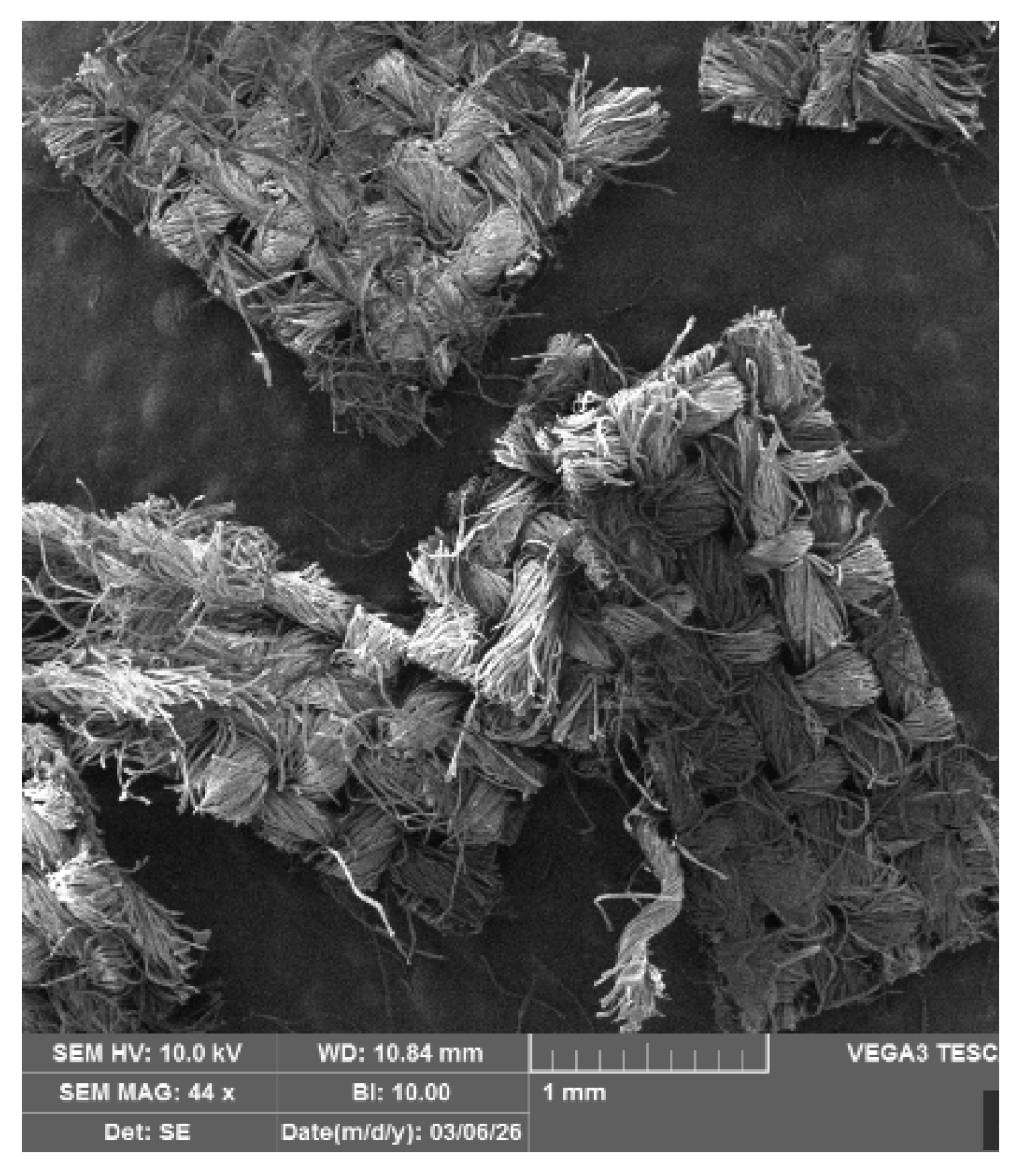	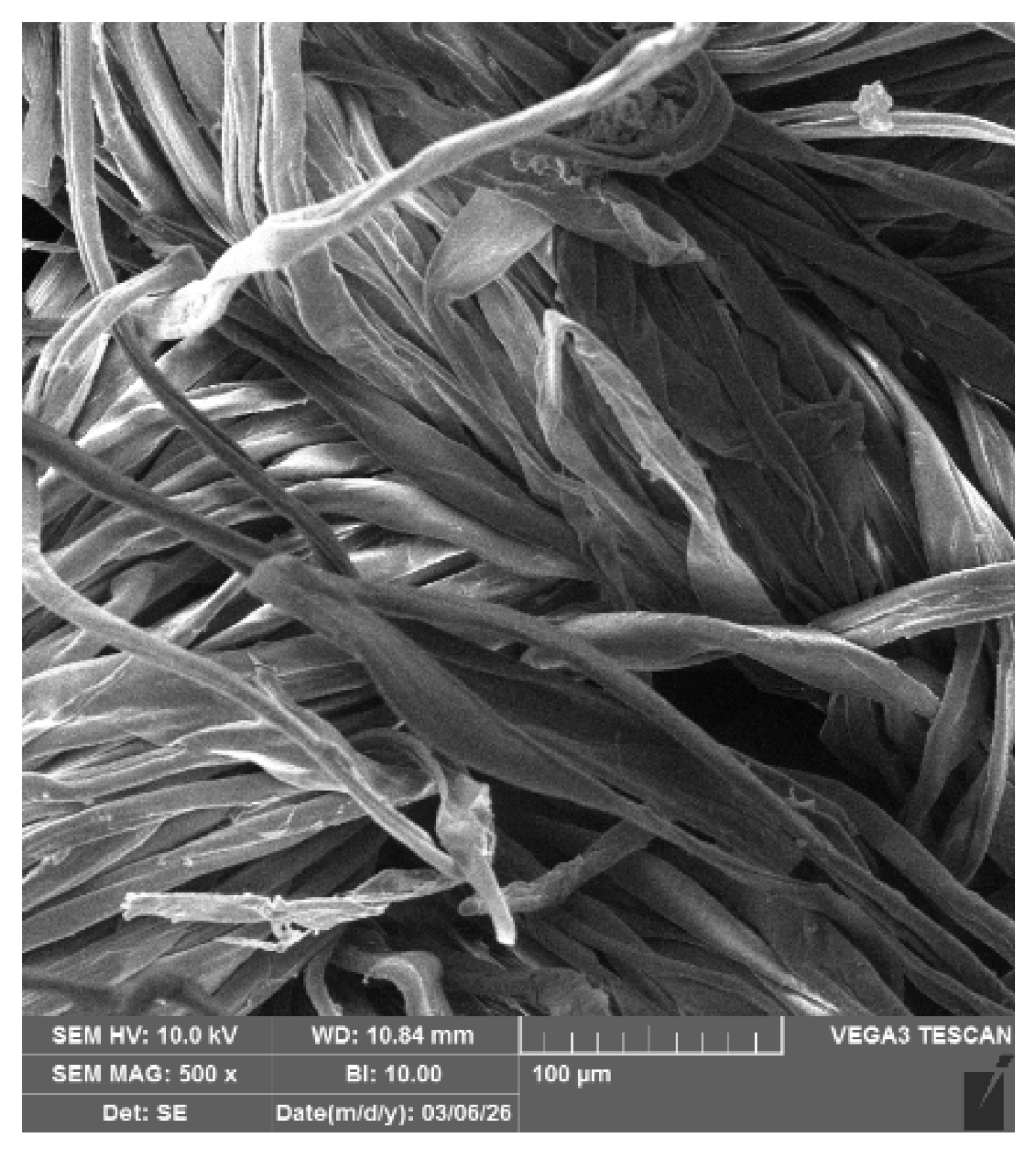	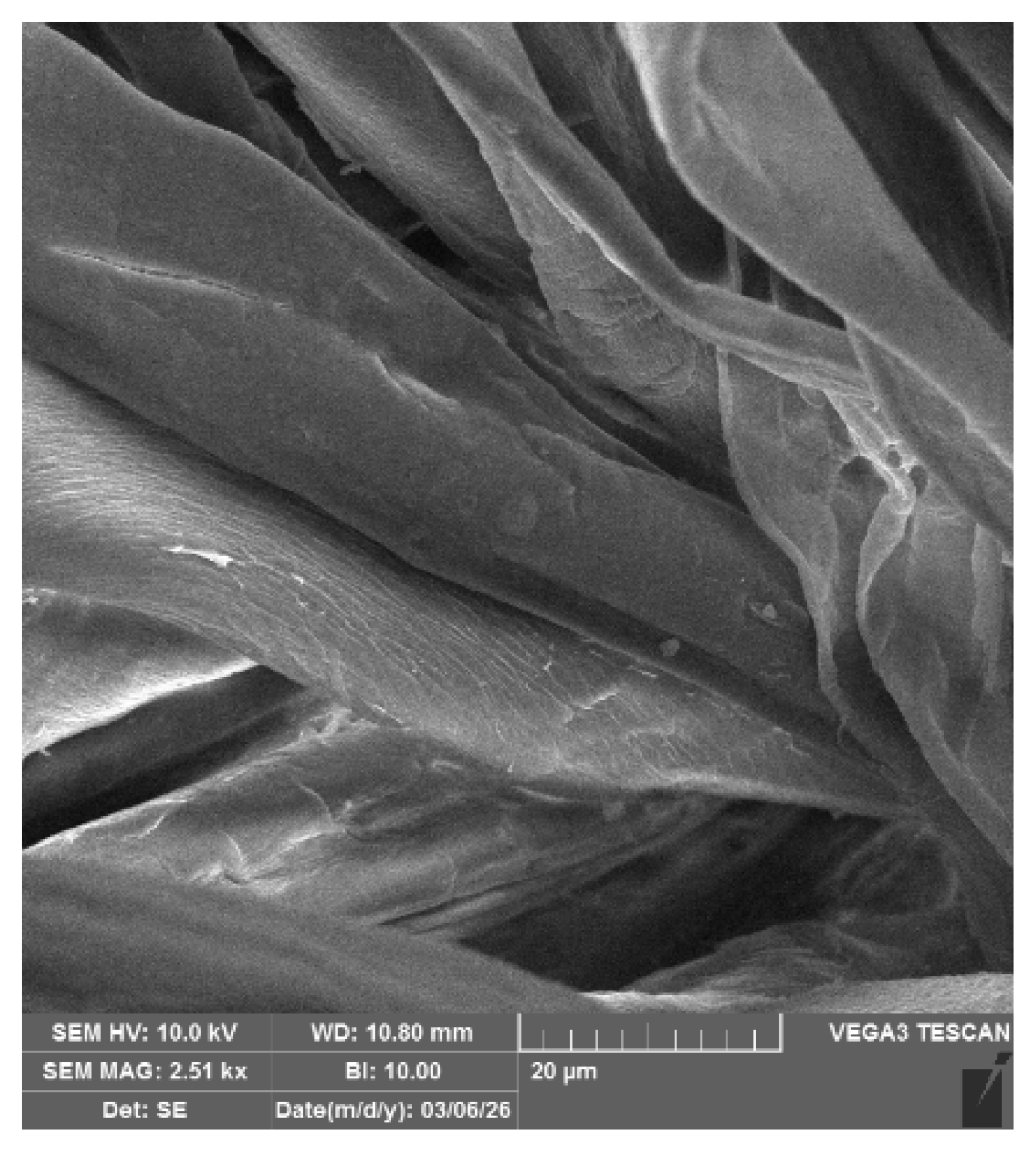
2 c	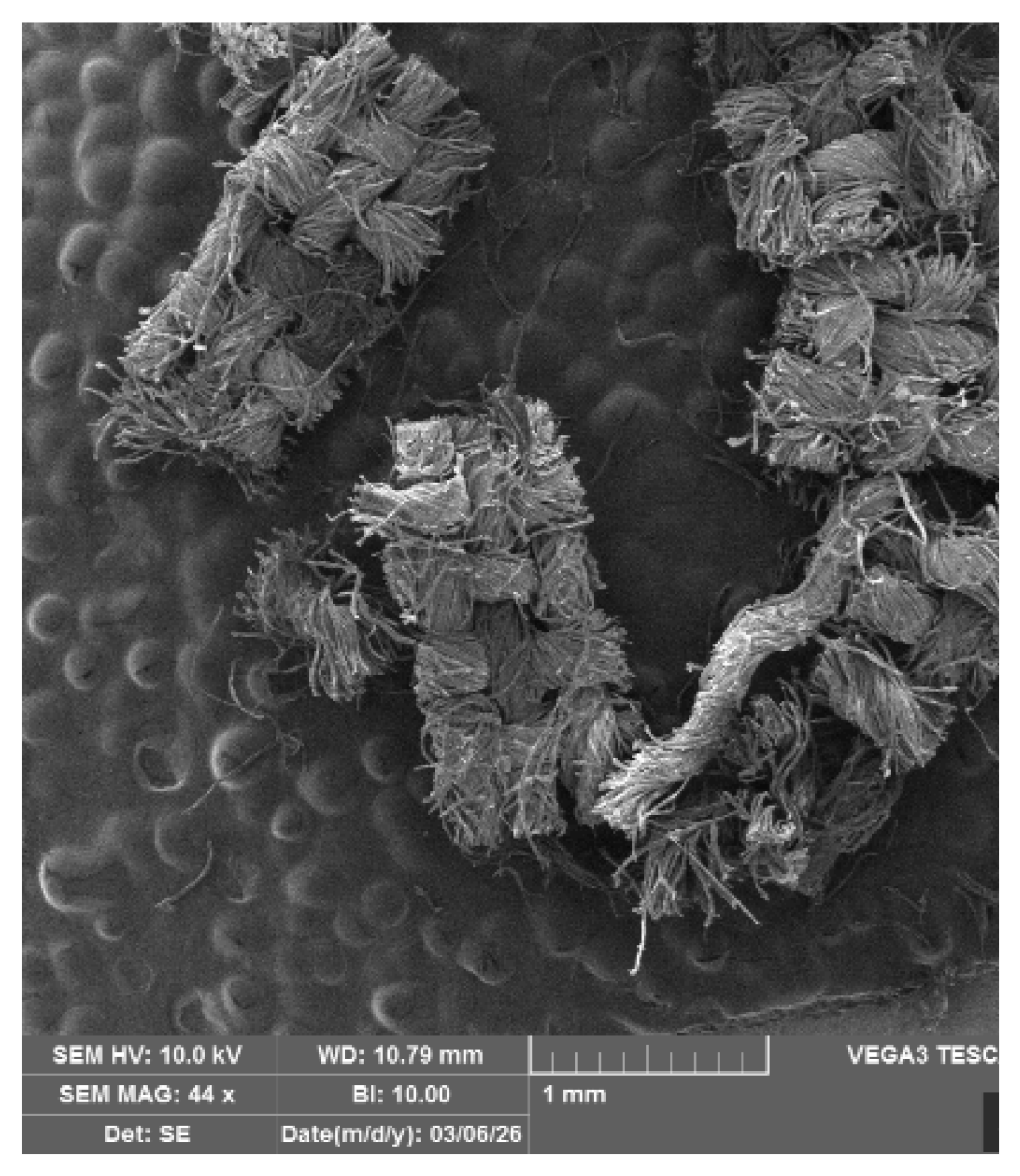	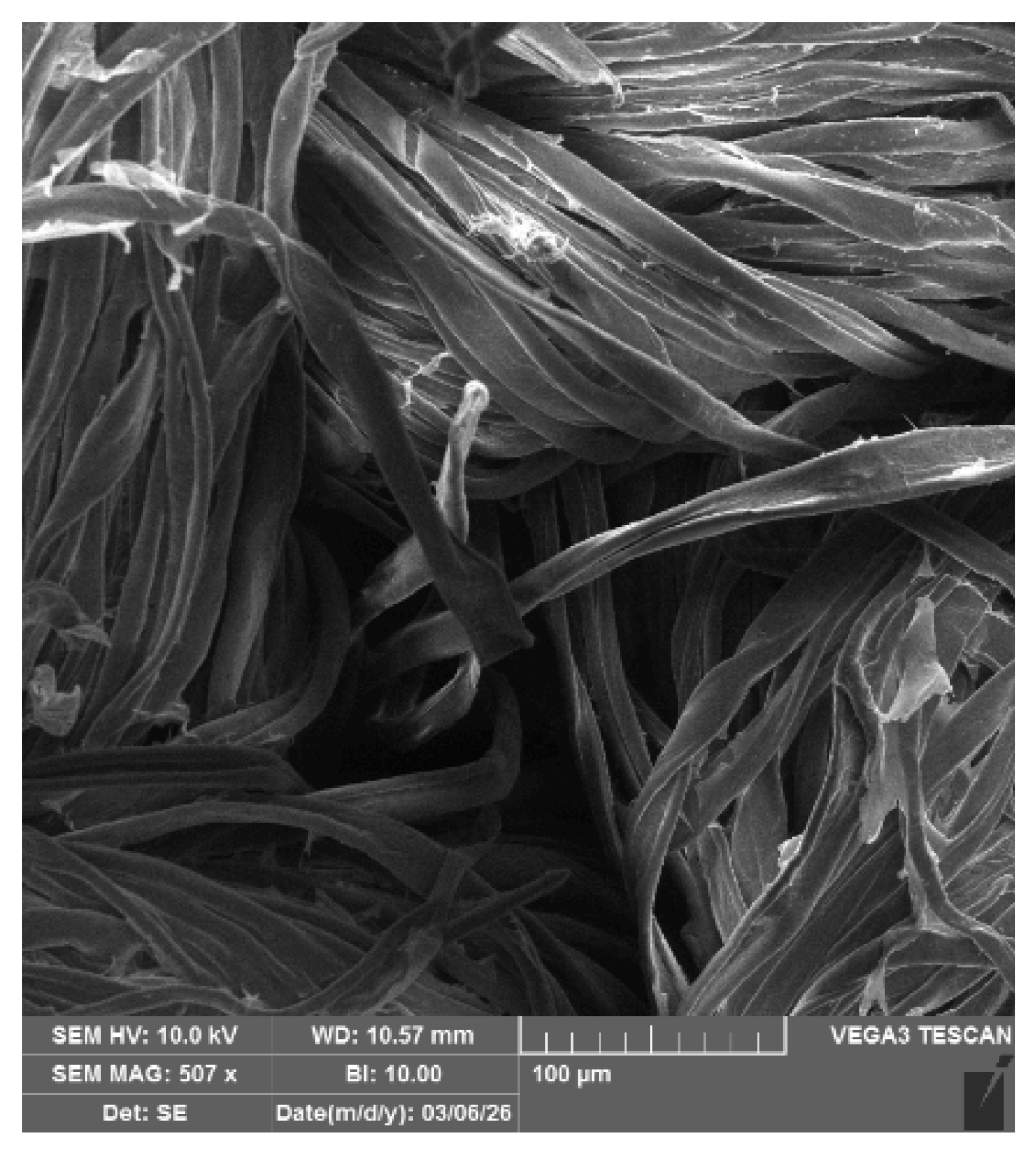	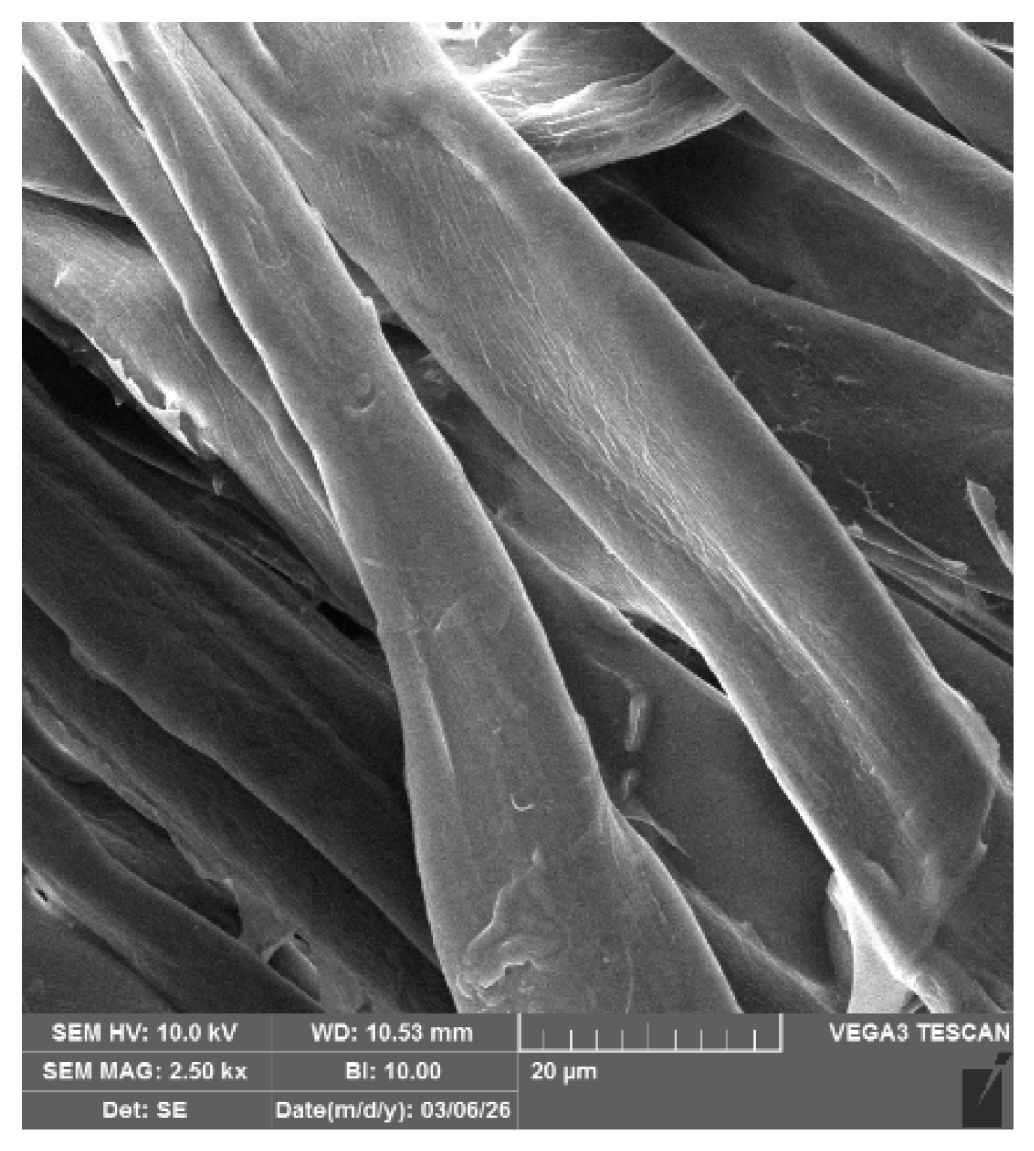
B	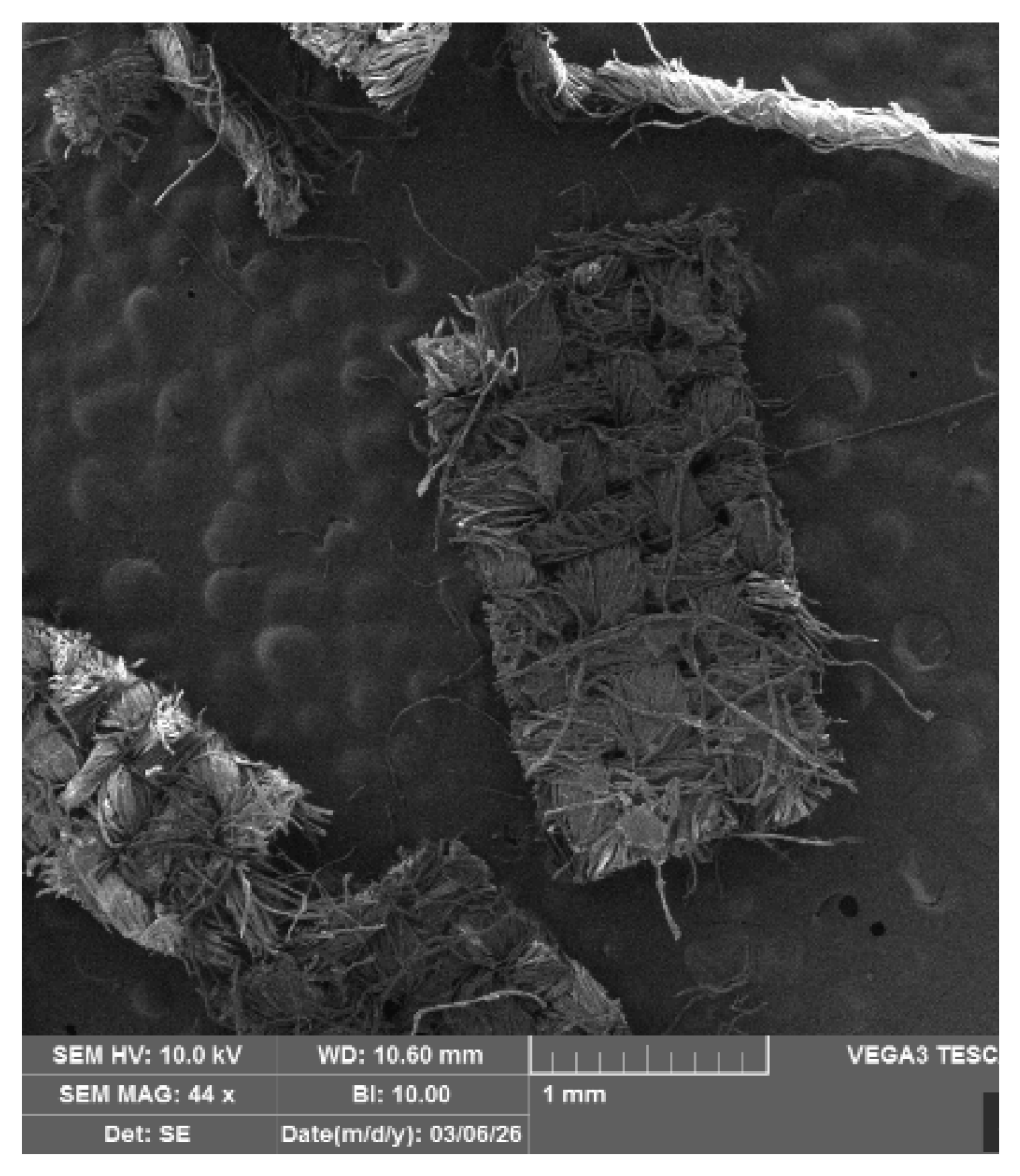	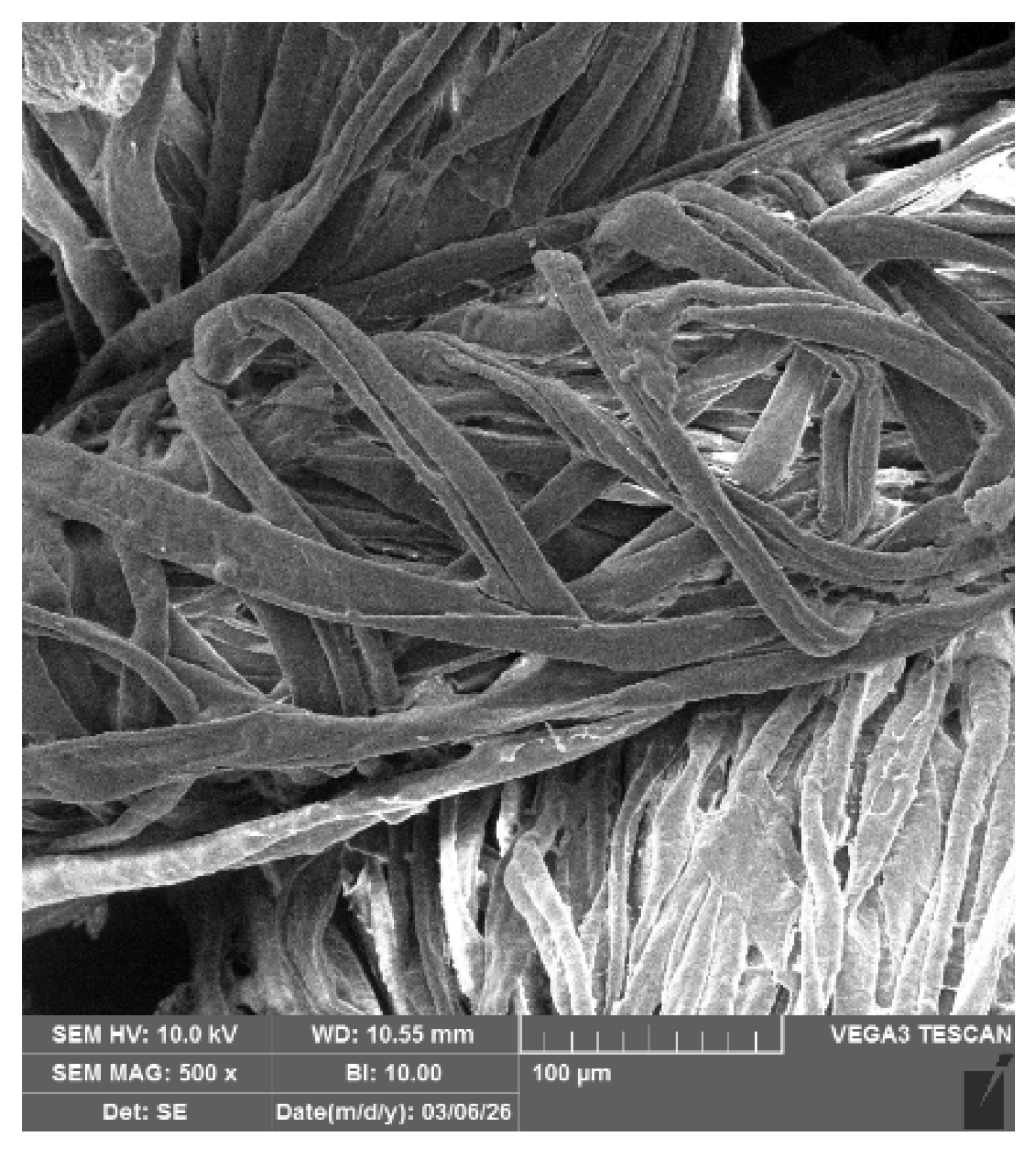	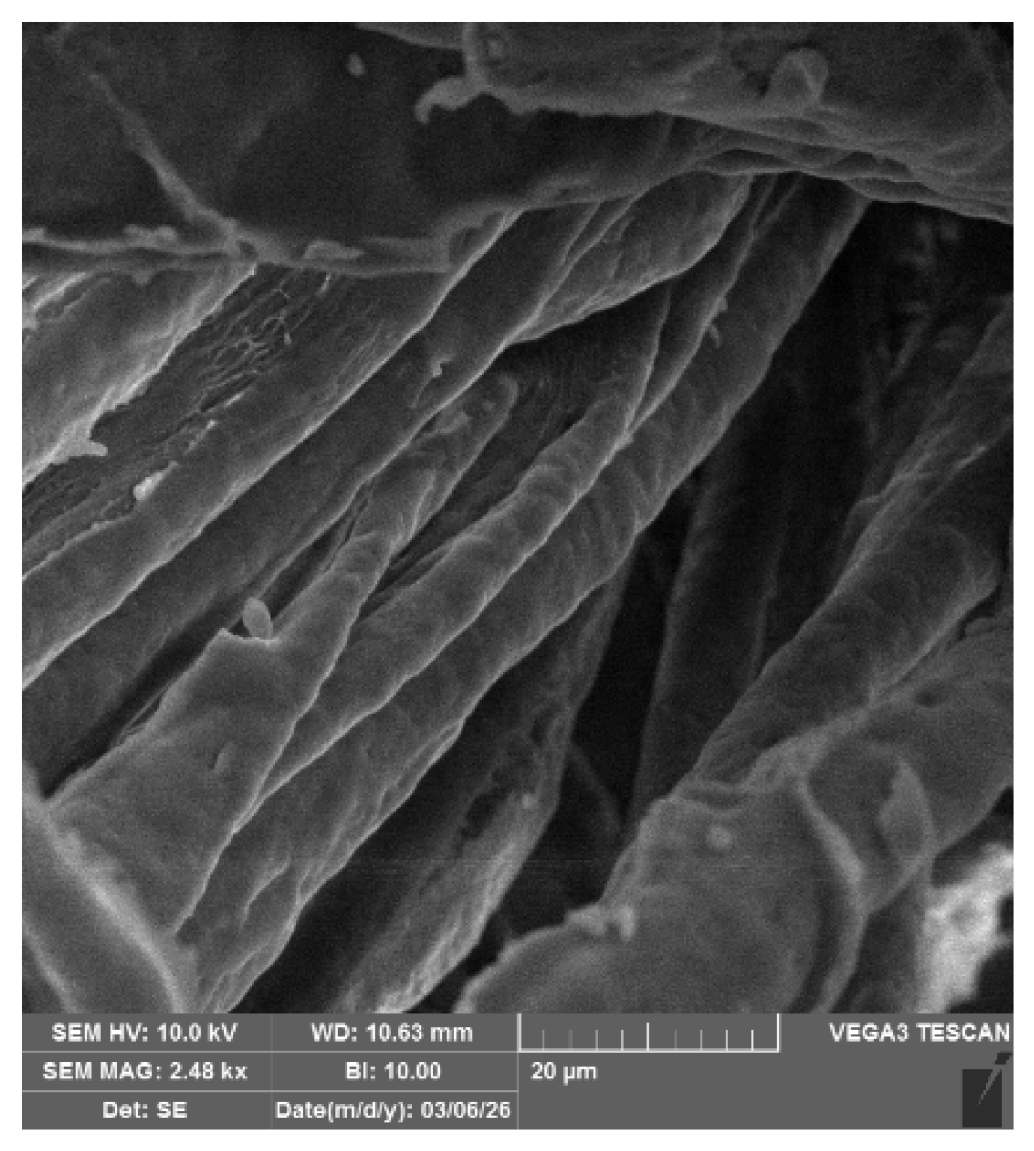
C	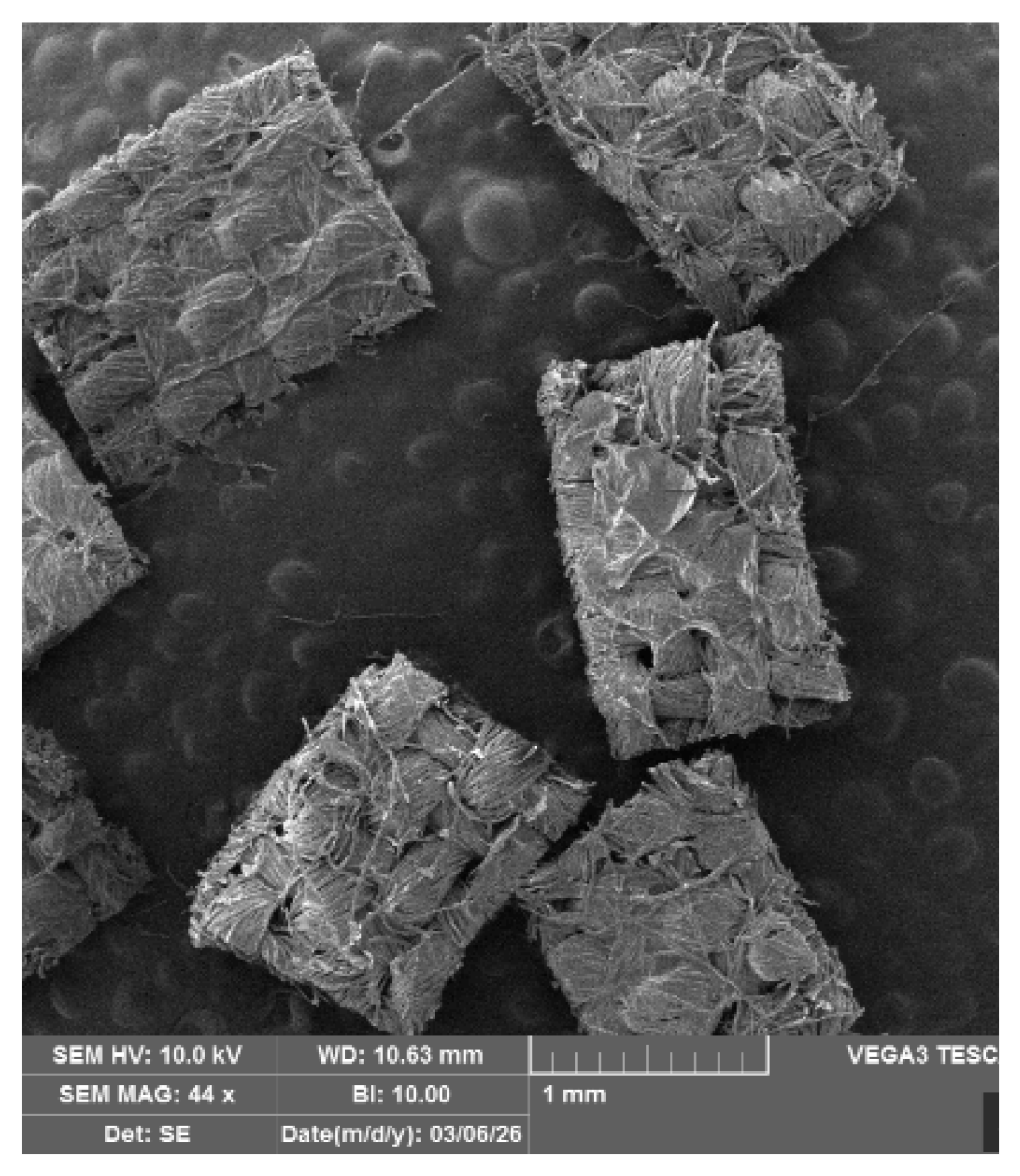	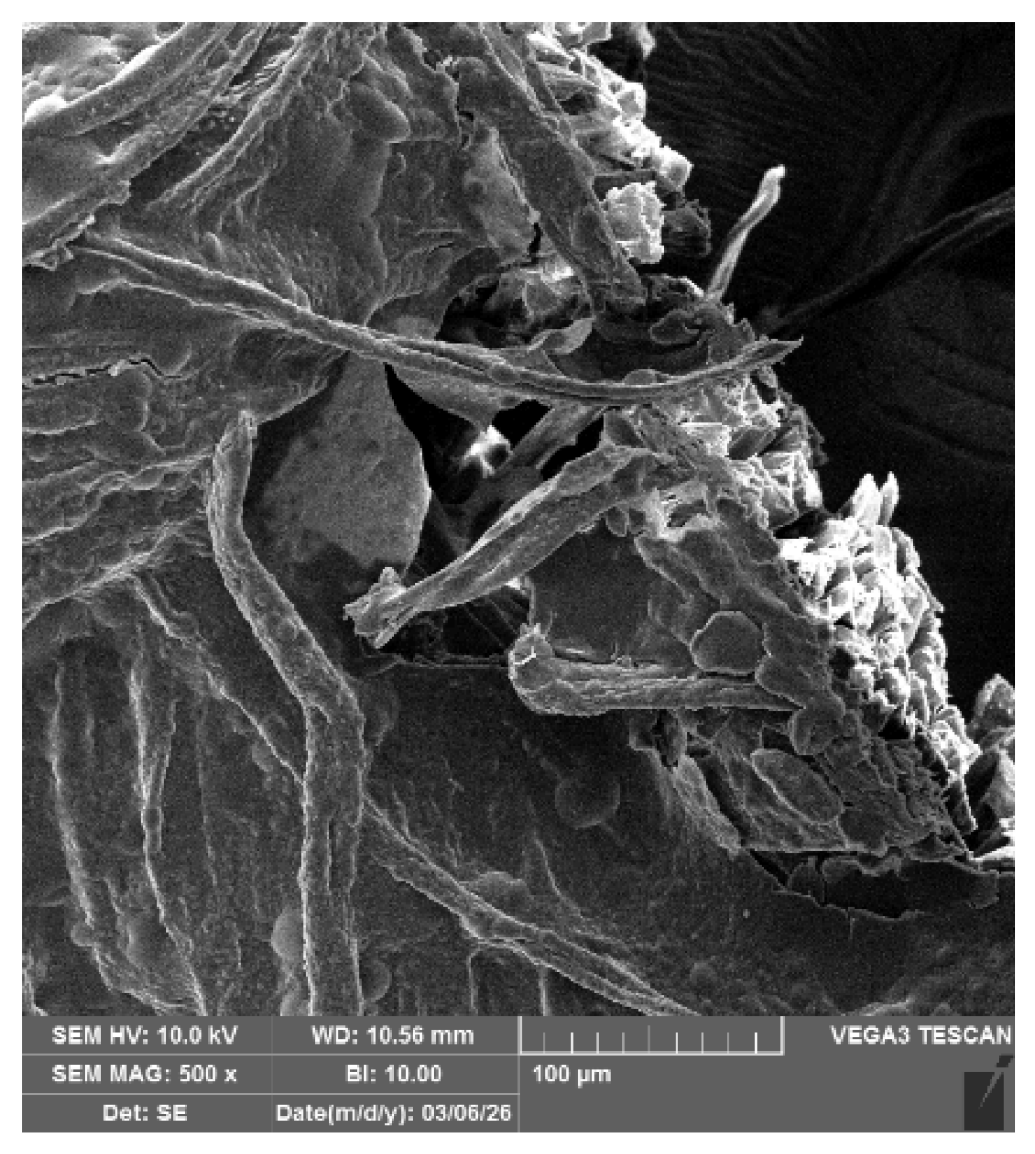	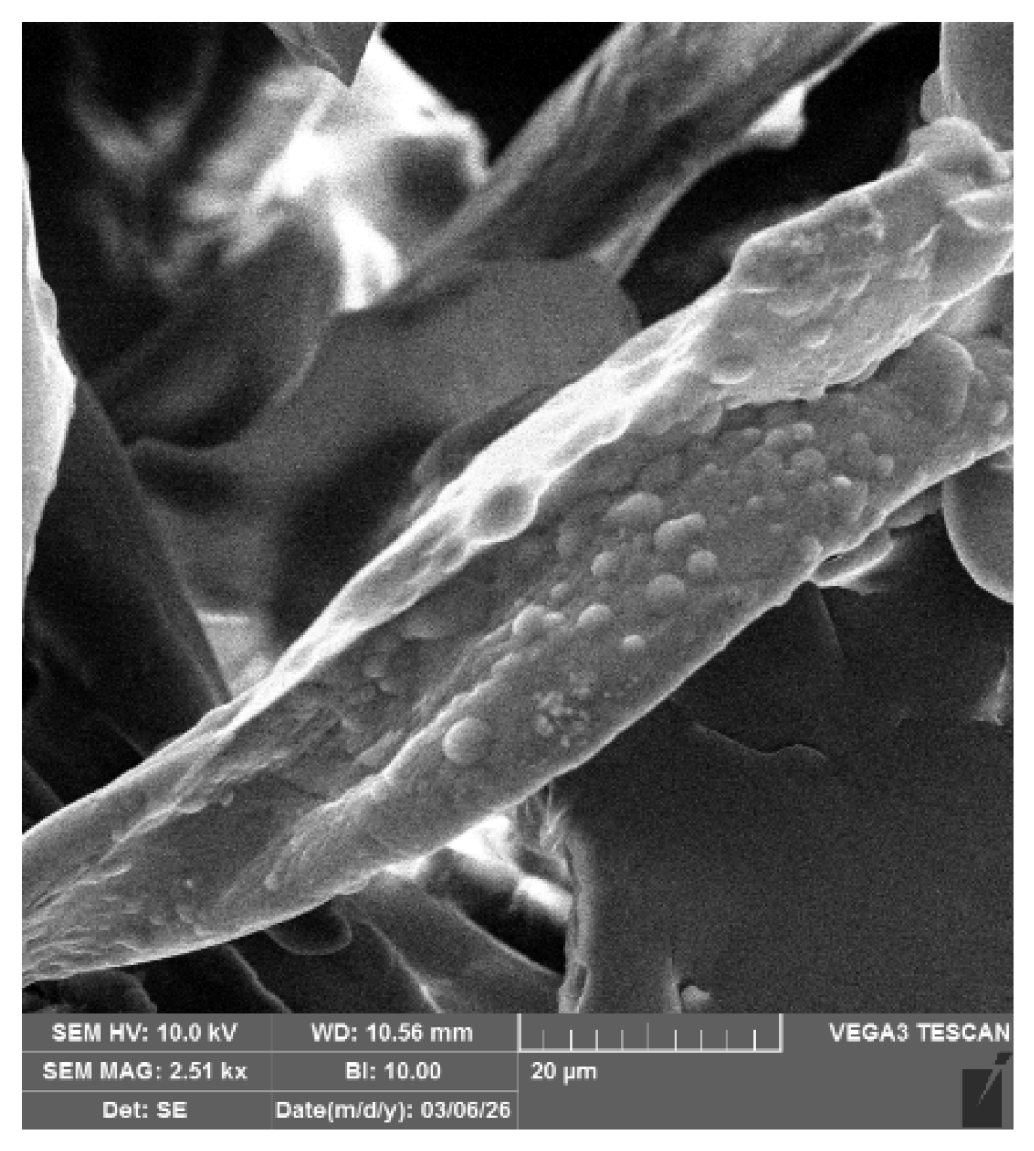
	2500×, emulsification on microlevel		
	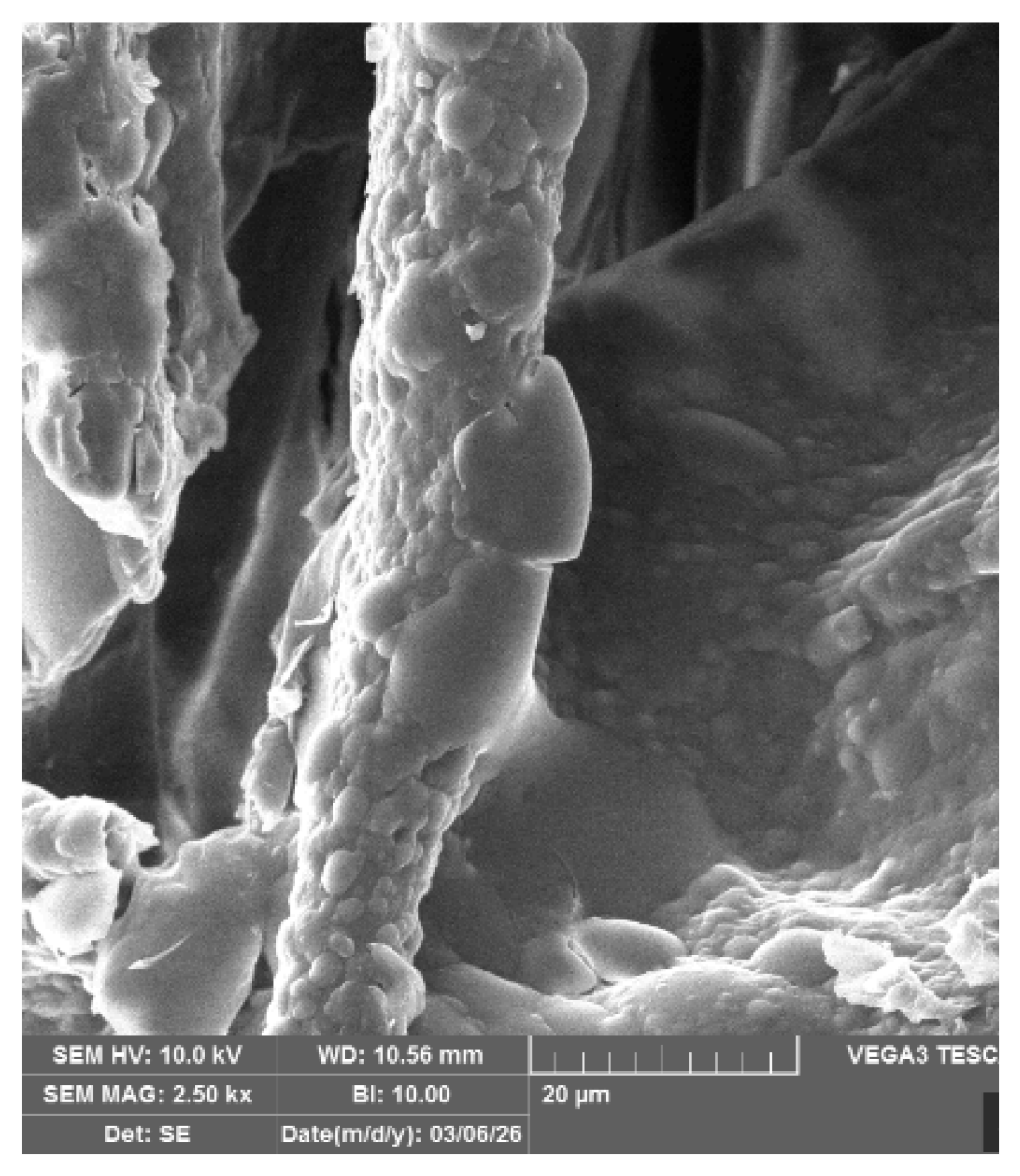	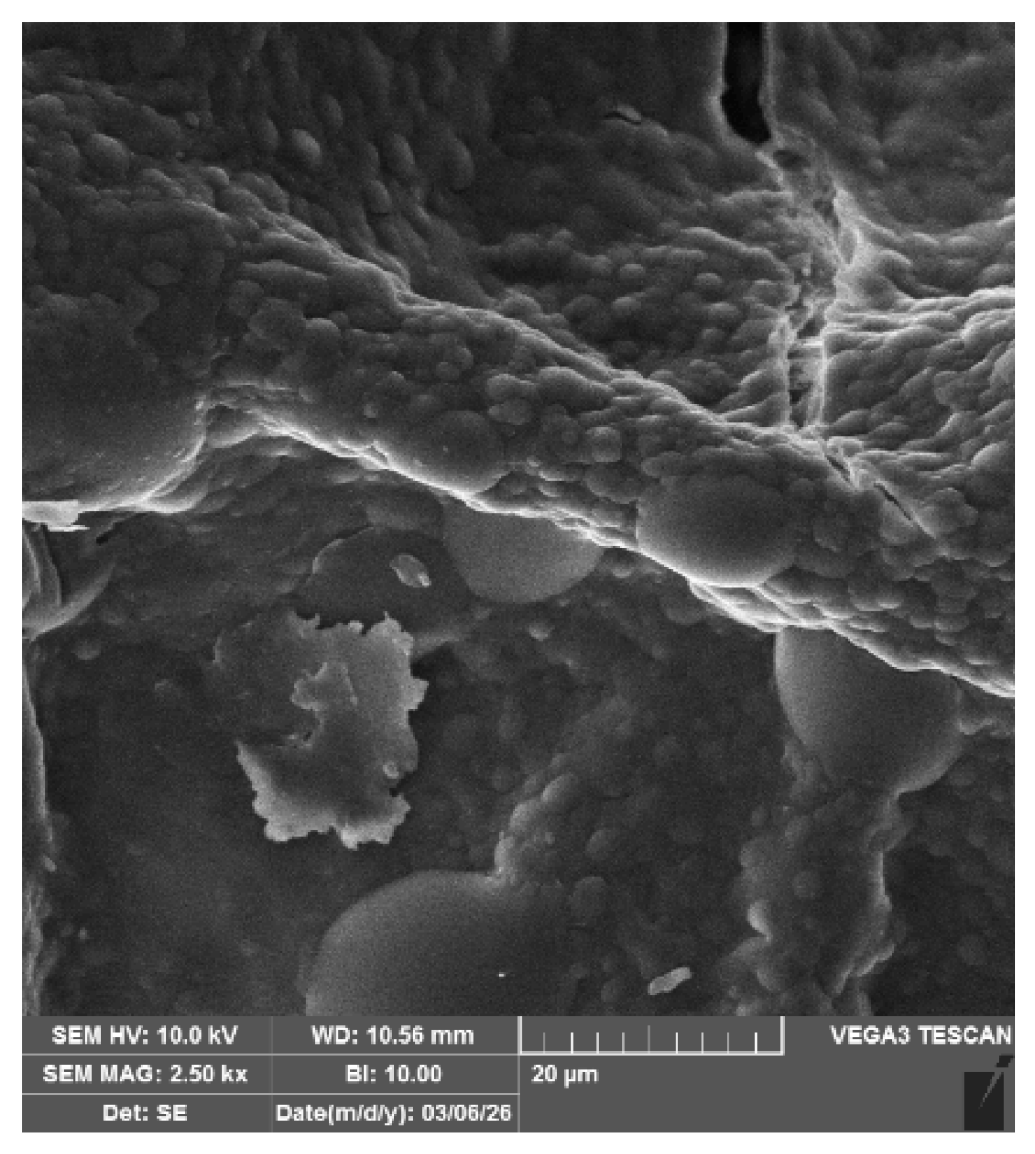	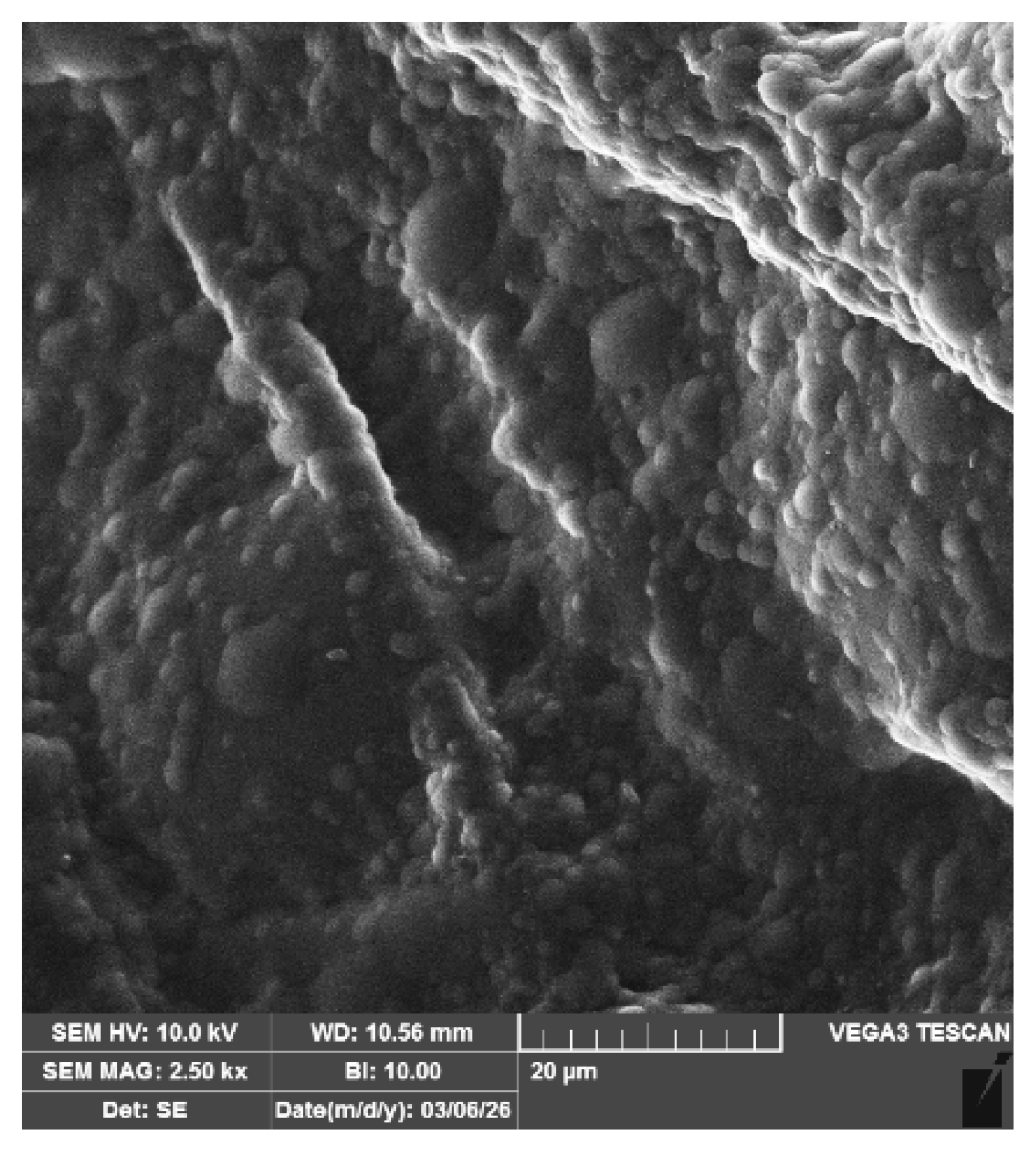

## Data Availability

Detailed information on the project and the supporting data is available at the web page: https://www.fkit.unizg.hr/CoRe4Pharm (accessed on 20 May 2026).
